# Wideband circularly polarized leaky wave rectenna

**DOI:** 10.1038/s41598-025-34021-3

**Published:** 2026-01-29

**Authors:** Nesma Mohamed, Nermeen A. Eltresy, Basem E. Elnaghi, Ahmed Magdy, Esmat A. Abdallah

**Affiliations:** 1 Electrical Engineering, Faculty of Engineering, Suez Canal University, Ismailia, 41522 Egypt; 2 Center of Nano Electronics & Devices, The American University in Cairo, Cairo, 11835 Egypt; 3 Communication and Electronics Department, Higher Valley Institute for Engineering & Technology, El Obour city, 11828 Egypt; 4 Microstrip Department, Electronics Research Institute, Cairo, 12622 Egypt

**Keywords:** Energy science and technology, Engineering

## Abstract

This paper presents a circular polarized rectenna based on a leaky wave antenna (LWA). Integrating circular polarization with leaky wave radiation greatly improves energy harvesting by minimizing polarization losses and extending the spatial range of captured RF signals. The LWA exhibits rapid frequency-dependent beam scanning, which is efficient by enabling harvesting RF power at different directions based on the received power direction. The proposed rectenna consists of LWA array integrated with a rectifier circuit and is designed to harvest RF power at the 5G midrange band. The implemented LWA has wide beam scanning angle from − 21 to 29^o^ with a high gain value of 9.8 dBi at 5.3 GHz. A Rectifier circuit correlated with a matching circuit is implemented. The designed matching circuit is based on a wideband compression network to compress the variation ratio of the input impedance. The results of the matched rectifier circuit show that the implemented circuit can operate from 4.1 to 5.5 GHz with effective impedance matching and stable wideband performance. The rectifier circuit is designed using an SMS7630 Schottky diode with a low turn-on voltage. The LWA is fabricated, its parameters are measured, and compared with simulated results. Then the LWA is used in the receiving mode, integrated with the rectifier circuit, and measured. The proposed rectenna obtained a maximum measured DC output voltage of 1.2 V with conversion efficiency of 53.8% at 4.2 GHz with received power of 0 dBm.

## Introduction

The growing need for energy-efficient and sustainable energy solutions has driven extensive research into harvesting radio frequency (RF) energy for supplying energy to low-power electronic devices, such as wireless sensor networks, Internet of Things (IoT) nodes, and biomedical implants^1–3^. RF energy could be found in the modern wireless environment, and it is converted into usable DC power through rectifying antennas (rectennas), which typically consist of an antenna for energy capture and a rectifier circuit for AC-to-DC conversion^4^. Despite significant advancements in rectenna design, existing solutions often suffer from polarization mismatch, limited angular coverage, and insufficient power conversion efficiency, thereby restricting their real-world applicability. To address the growing demand for versatile energy solutions, recent research has explored integrated cooperative harvesters designed to scavenge hybrid energy, enabling their deployment in a wider range of application scenarios^5–9^. Traditional rectennas predominantly employ linearly polarized antennas, which are inherently sensitive to the orientation of incoming RF waves. As ambient RF signals are often randomly polarized or vary in polarization due to multi-path effects, linearly polarized rectennas exhibit inefficient energy reception when the incident wave’s polarization does not align with the antenna’s polarization axis^10^.

Alternatively, circularly polarized (CP) antennas offer inherent polarization diversity, allowing efficient energy capture regardless of the wave’s orientation. For instance, a study demonstrated that a CP rectenna array system could mitigate polarization mismatch, thereby enhancing the harvested power^11^. To effectively mitigate polarization mismatch in environments characterized by unknown polarization, the utilization of CP antennas and dual-polarization antennas are recommended. Among these, circular polarized antennas are particularly well-suited for beamforming directed wireless power transfer (WPT) applications that involve a dedicated power source^12–17^. Mattsson et al.^13^ present a dual-band rectenna operating at 2.4 GHz, 5.5 GHz, and to improve the output voltage, a full-wave voltage doubler rectifier is implemented for each polarization. Furthermore, Surender et al.^14^ describe a CP dielectric resonator rectenna operating at 5.8 GHz, achieving a power conversion efficiency (PCE) of 70.5% at an input power level of 5.75 dBm. In addition, Du et al.^16^ propose a broadband CP rectenna, achieving a PCE of 76% at 14 dBm, that integrates a wideband rectifier based on a resistance compression network (RCN) and a wideband circular polarized antenna employing a phase shifter.

Furthermore, conventional rectennas utilize fixed-beam antennas, limiting their ability to capture spatially distributed RF energy. The PCE of rectennas is often limited by the receiving antenna gain, which limits the RF power supplied to the rectifier circuit. This limitation is particularly pronounced in far-field RF energy harvesting applications, where increased distance from the source reduces incident power density. Current researches^18,19^ emphasize the need for high-gain receiving antennas to compensate for diminished power density. While directional antennas can improve received RF power, their narrow beamwidth restricts angular coverage. In practical scenarios, the indeterminate positioning between the RF source and energy harvester introduces challenges due to the incident angle-dependent PCE^20^. In^21–26^ the trade-off between antenna gain and angular coverage was investigated. Eid et al.^24^ presented a wide-angle rectenna employing a Rotman lens for 5G millimeter-wave energy harvesting at 28 GHz, obtaining a realized gain of 17 dBi per port with 110⁰ of angular coverage. However, the Rotman lens has significant drawbacks, including size and insertion loss. Furthermore, a retrodirective rectenna array operating at 5.8 GHz, as introduced by Ren et al.^26^ exhibited a low gain of 5.89 dBi with wide beamwidth.

A leaky wave antenna (LWA) is a type of antenna that radiates electromagnetic energy gradually along its structure, rather than only at its ends. This leakage of energy allows the antenna to generate a traveling wave that radiates in a specific direction, making it useful for applications requiring directional beamforming^27^. LWAs have garnered significant attention in modern electromagnetic and antenna design due to their ability to achieve high directivity, beam steering, and frequency-dependent radiation characteristics. These antennas operate by utilizing traveling waves that progressively leak energy into free space, making them ideal candidates for radar systems, wireless communications, and satellite technology applications. The conventional LWA consists of slotted rectangular waveguides, where the fundamental principle involves electromagnetic waves propagating along the waveguide structure and radiating into free space as fast waves^27^. While conventional planar LWAs rely on slow-wave propagation within their guiding structures, the incorporation of periodic perturbations allows for the excitation of spatial harmonics, thereby enabling backward-to-forward radiation characteristics. The inherent open stopband characteristic of conventional LWAs^28–30^ presents a challenge in achieving high-efficiency broadside radiation. Current LWAs highlights significant advancements in their design, including the use of periodic structures, composite right/left-handed transmission lines, and metasurfaces to manipulate wave radiation characteristics. High-efficiency broadside radiation can be achieved through an antenna structure based on asymmetrical two-sided periodic depth-modulated metallic grooves^31^.

Xu et al.^32^ presented optimal broadside radiation characteristics based on double layers of anti-phase-inversion surface plasmons polaritons (SPPs) waveguides. Additionally, a single-layer LWA based on meandered SPP cells exhibits stable scanning beams with a consistent gain variation of less than 2.5 dB across a broad − 10 dB reflection bandwidth^33^. Beyond periodic modulation integrated directly into the SPP waveguide structure, the introduction of periodically loaded metallic patches in proximity to the waveguide enables modulation and coupling with the transmission line, leading to efficient radiation characteristics^34^. Zhang et al.^35^ reported a CP LWA based on a SPP transmission line, incorporating two rectangular patch arrays configured with a 90° phase shift to obtain CP. This LWA demonstrated matching bandwidth exceeding 33% and a 3-dB axial-ratio bandwidth of 15%. Within the frequency range of 5.5 to 6.4 GHz, the antenna has gain of 10 dBic. Chen et al.^36^ utilized a substrate-integrated waveguide shaped like a benzene ring, featuring partially reflecting wall vias. Ma et al.^37^ proposed an innovative planar Fabry–Perot resonator antenna featuring CP, frequency beam scanning capability, and improved gain performance. The antenna consists of a two-layer structure. The lower layer comprises a CP substrate-integrated waveguide beam-scanning LWA, serving as the feed and ensuring a fixed radiation pattern across the operating band. Additionally, a partially reflecting surface layer is integrated to improve gain, achieved through an array of metal patches printed on both sides of substrate. SPPs have emerged as a significant research area in electromagnetics and plasmonics, offering novel solutions for wave confinement, guiding, and radiation control at microwave and terahertz frequencies SPPs are electromagnetic (EM) waves that propagate along the edge between two media possessing different signs of permittivity, exhibiting exponential decay within both media. Periodic structures can be employed to realize artificial equivalent negative permittivity in the microwave frequency band^38^. Spoof SPPs (SSPPs), a class of artificial periodic structures, emulate the wave-confining properties of natural SPPs within a compact circuit size. The conversion structure facilitates a wide passband by transforming the electromagnetic mode from the SSPP state to a spatial mode^39^. The inherent properties of SSPP waveguides, including high field confinement, tunable dispersion, low ohmic losses, and flexible planar geometries have enabled their integration with diverse design methodologies for the realization of various microwave RF devices, including power dividers^40^, filters^41^, phase shifters^42^, and antennas^43^. A dual-beam scanning antenna for 5G applications, utilizing a slow-wave transmission line based on SPP modes operating below a specified cutoff frequency for excitation was presented in^44^.

LWA-based rectennas remain unexplored in the context of circular polarization, which could further enhance harvesting efficiency and reliability in dynamic wireless environments. In this paper LWA antenna fed with SPP-TL is applied for RF-EH due to their high gain, frequency-dependent beam scanning capability, which allows for wider angular coverage and improve energy collection over a broad spatial region. The designed LWA has a wide operating frequency bandwidth that extends from 2 to 8 GHz for EH at Wi-Fi 2.4, LTE 2600, WLAN, WiMAX and the expected 5G mid-bands.

Wide-angle CP radiation of proposed CPLWA array combined with voltage doubler harvesting, is clearly clarified when comparing our results with the previously published. Whether for CP LWA or CP rectennas. The structure of the SSPP fed is one of the techniques that was also used for obtaining wide bandwidth. These results are confirmed using simulation with different ready-made software packages and measurements after fabricating the rectenna. The paper is organized as the following: first the design of the CP LWA is explained starting with the design of the SPP-TL, indicating the shape and the parameters of the SPP-TL unit cell. Then the radiator element is implemented which after that are loaded on the SPP-TL to form the CP LWA array. The array is fabricated and measured. In the following sections the rectifier, matching circuit is implemented, explained, fabricated, integrated with the CP LWA array and measured. Final section concludes all the work.

## CP-leaky wave antenna design

**Fig. 1:**
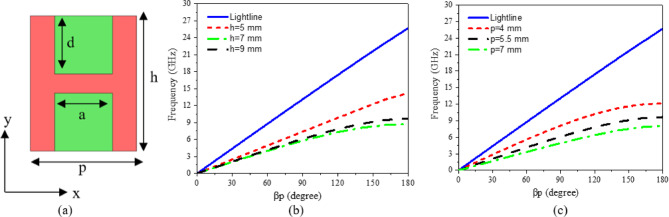
The geometry of H shape unit cell and its dispersion curve with different values of h and p; (**a**) Image of proposed H shape unit cell, (**b**) effect of height h, and (**c**) effect of periodicity p.

**Fig. 2:**
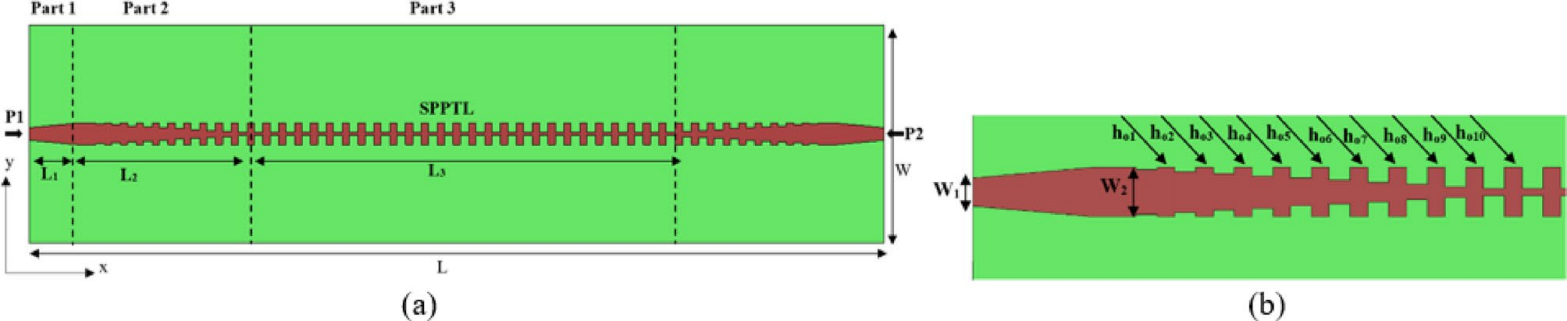
The structure of spoof transmission line (STL).

**Fig. 3:**
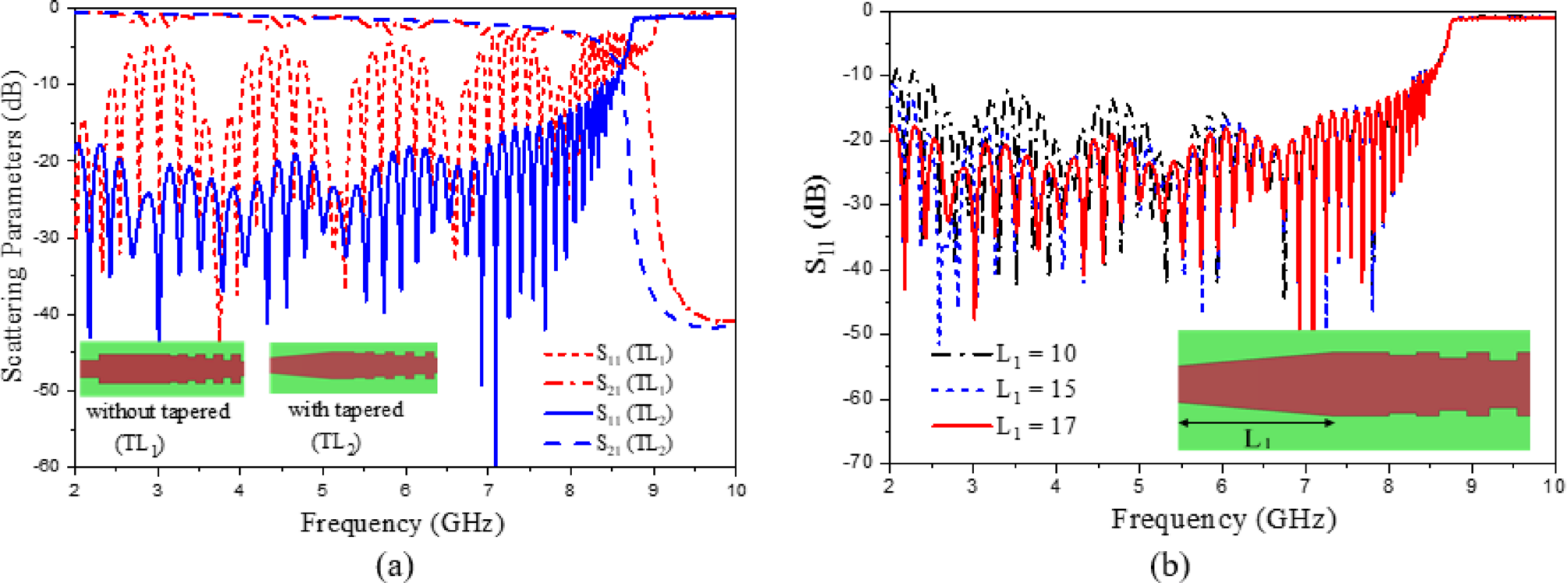
(**a**) Comparison of STL without and with tapered according to scattering parameters, and (**b**) reflection coefficient variation with frequency for different values of L_1_.

The proposed LWA consists of CP radiating elements fed by a spoof transmission line (STL). The STL is designed using a unit cell with an H-shaped corrugated structure with periodicity (*p* = 5.5 mm), height (h = 7 mm), depth (d = 3 mm), and width (a = 3 mm), as illustrated in (Fig. 1a). The red region denotes the metallic layer, while the green region denotes the dielectric substrate. The backside of the unit is entirely covered by a metallic layer, serving as the ground plane. In this design, the dielectric substrate is selected as Rogers Ro4003C, with copper used for the metallic layer. The substrate has $\:{\epsilon\:}_{r}$= 3.38, and $\:tan\delta\:=$ 0.0027. The substrate thickness is 1.52 mm. Equation (1) defines the dispersion relation for the STL^45^.

1 $$\:{K}_{SPP}={k}_{o}\sqrt{1\:+\frac{{a}^{2}}{{p}^{2}}}\:{tan}^{2}\left({k}_{o}h\right)$$

where $$\:{k}_{o}$$ $$\:=$$ $$\:\raisebox{1ex}{$\omega\:$}\!\left/\:\!\raisebox{-1ex}{$c$}\right.$$ Represents the wave number in free space, c denotes light speed in free space, and a, p, and h correspond to the width, period, and height of the corrugated strips, respectively. The unit cell’s dispersion characteristics are analyzed using the Eigenmode solver in CST Microwave Studio^46^. As depicted in Fig. 1b, c, the dispersion curves progressively shift away from the airline, with the propagating mode consistently remaining below it. The periodicity p and groove height h of the H-shaped structure have a significant effect on the dispersion characteristics. Consequently, SSPPs operate within the slow-wave region. The H-shaped unit cell exhibits a cutoff frequency of 8.66 GHz. Since the proposed LWA is designed to operate within the frequency range of 2 GHz to 8 GHz, the STL is suitable for integration into the current antenna design. The schematic representation of the proposed STL is depicted in (Fig. 2). The STL is divided into three sections: part 1 is a 50 Ω microstrip transmission line. Part 2 serves as a transition region enabling gradual mode conversion between part 1 and part 3. Part 3 is a periodic structure composed of H-shaped SPP units. To facilitate impedance matching between the 50 Ω microstrip line and the high-impedance waves of the STL, gradient corrugation grooves are employed at the interface, as illustrated in (Fig. 2b). Additionally, a linear tapered section is incorporated to connect the feeding line and improve return loss, as shown in (Fig. 2b). This compares the simulated S-parameters for the STL without and with a tapered section. The STL without a tapered section exhibits significantly degraded performance due to intrinsic momentum and impedance mismatches. Furthermore, inserting the linear tapered section significantly improves the return loss, that can be seen in (Fig. 3a). Furthermore, the effect of the tapering length of Part 1 (L_1_) is shown in (Fig. 3b). It can be seen that the return loss is directly affected by the length (L_1_), and increasing the tapering length improves the matching. The optimal performance is achieved at L_1_ = 17 mm. The final detailed dimensions obtained from the 3D simulations are presented herein: L = 297 mm, W = 70 mm, L_1_ = 17 mm, L_2_ = 60 mm, L_3_ = 143 mm, W_1_ = 4 mm and W_2_ = 7 mm.

**Fig. 4:**
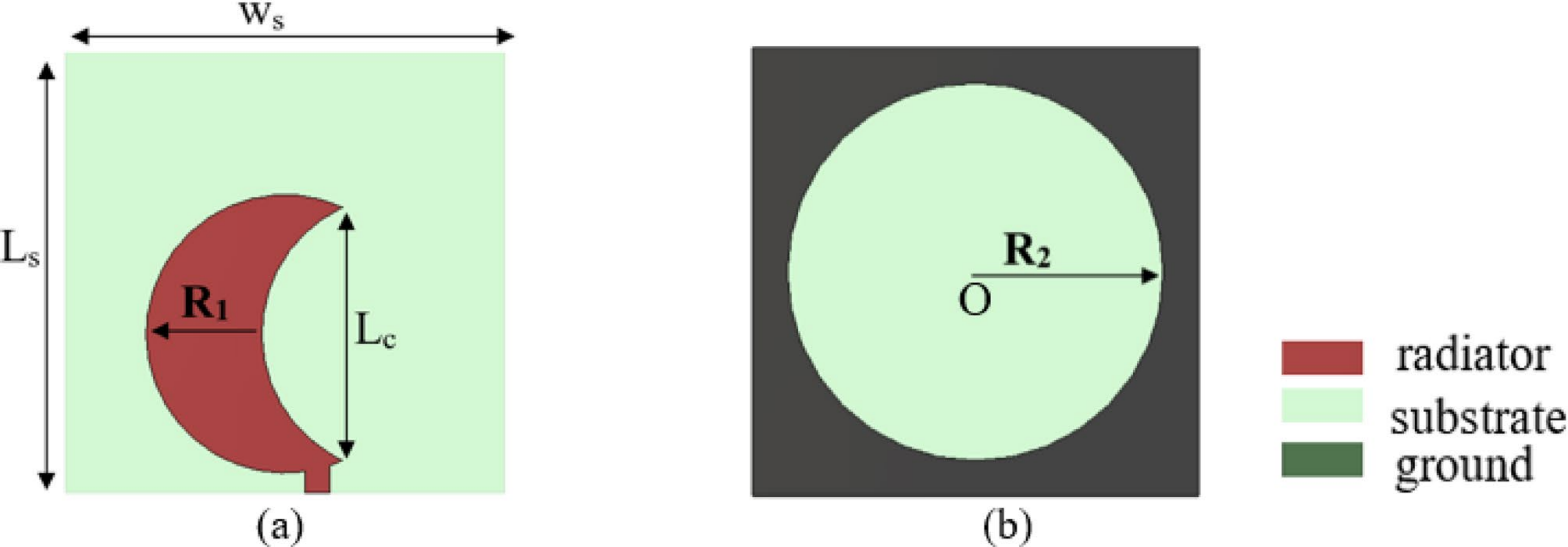
The structure of the Radiating element; (**a**) top view, (**b**) bottom view.

The radiating element antenna is implemented using same substrate of unit cell. The detailed structure of the radiating element antenna is illustrated in (Fig. 4). The antenna is designed and simulated using Ansys HFSS^47^ and CST Microwave Studio^48^. It comprises a crescent-shaped monopole antenna at the top and a circular-shaped slot in the ground plane at the bottom^49^. A 50 Ω microstrip feedline, with length L_F_ = 2.4 mm and width W_F_ = 1.5 mm, is employed to feed the antenna. The monopole antenna comprises two concentric circles with an identical radius denoted by R_1_ = 8.4 mm, which are subtracted from one another with a centre-to-centre separation distance specified separation distance L_C_ = 15.27 mm. The antenna’s ground plane comprises a rectangular region with dimensions L_S_ × W_S_ = 26.6 × 26.6 mm², from which a centrally located circular-shaped structure is subtracted with a radius of R_2_ = 11.2 mm.

**Fig. 5:**
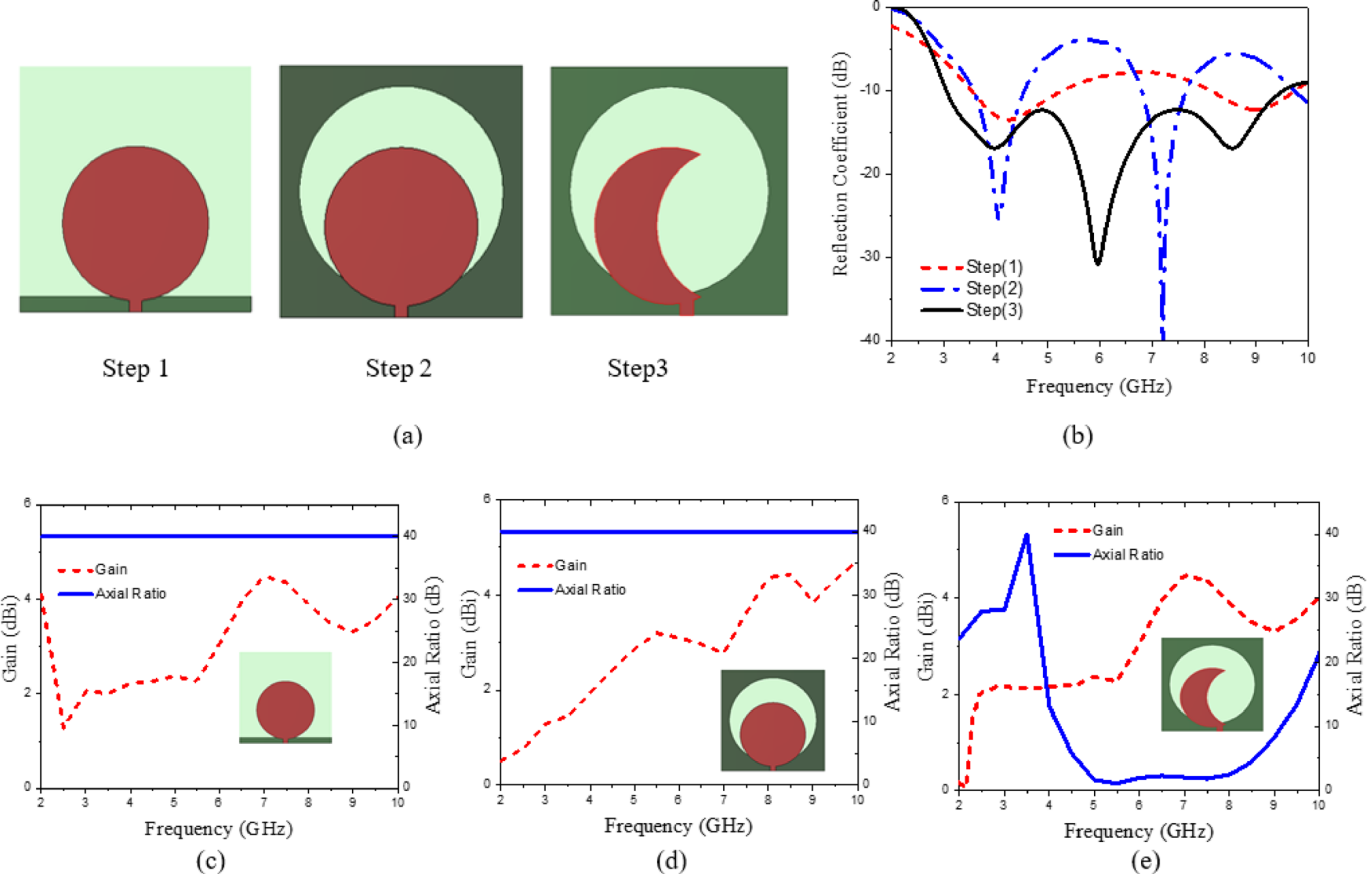
(**a**) The design steps of radiating element, (**b**) The corresponding reflection coefficients for each step, (**c**–**e**) gain and axial ratio variations versus frequency for each step.

The antenna was designed following the procedure steps outlined in (Fig. 5a), and the corresponding S-parameters at each design step are presented in (Fig. 5b). The initial step, as shown in (Fig. 5a), involves selecting a monopole circular patch antenna with a partial ground plane that has appropriate dimensions, which is known to exhibit broadband characteristics. The printed monopole antenna has an overall area of W_S_ × L_S_ = 26.6 × 26.6 mm². The initial length of the printed monopole antenna is determined^50^ using Eq. (2).

2 $$\:{L}_{S}=\frac{1}{4\:{f}_{r}\sqrt{{\epsilon\:}_{eff}}\sqrt{{\mu\:}_{o}{\epsilon\:}_{o}}}$$

**Fig. 6:**
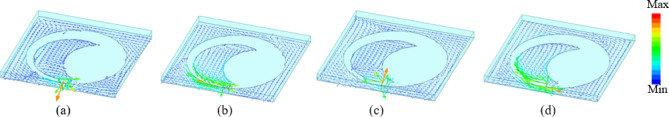
Surface current distribution of the antenna at 5 GHz at different phase angles. (**a**) 0◦, (**b**) 90◦, (**c**) 180◦, and (**d**) 270◦.

**Fig. 7:**
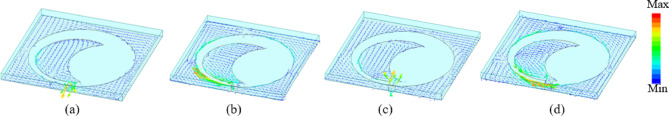
Surface current distribution of the antenna at 5 GHz at different phase angles. (**a**) 0◦, (**b**) 90◦, (**c**) 180◦, and (**d**) 270◦.

**Fig. 8:**
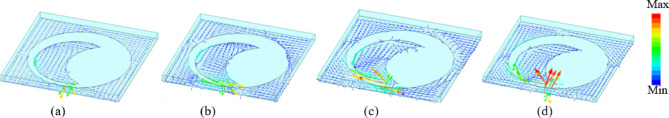
Surface current distribution of the antenna at 8 GHz at different phase angles. (**a**) 0◦, (**b**) 90◦, (**c**) 180◦, and (**d**) 270◦.

**Fig. 9:**
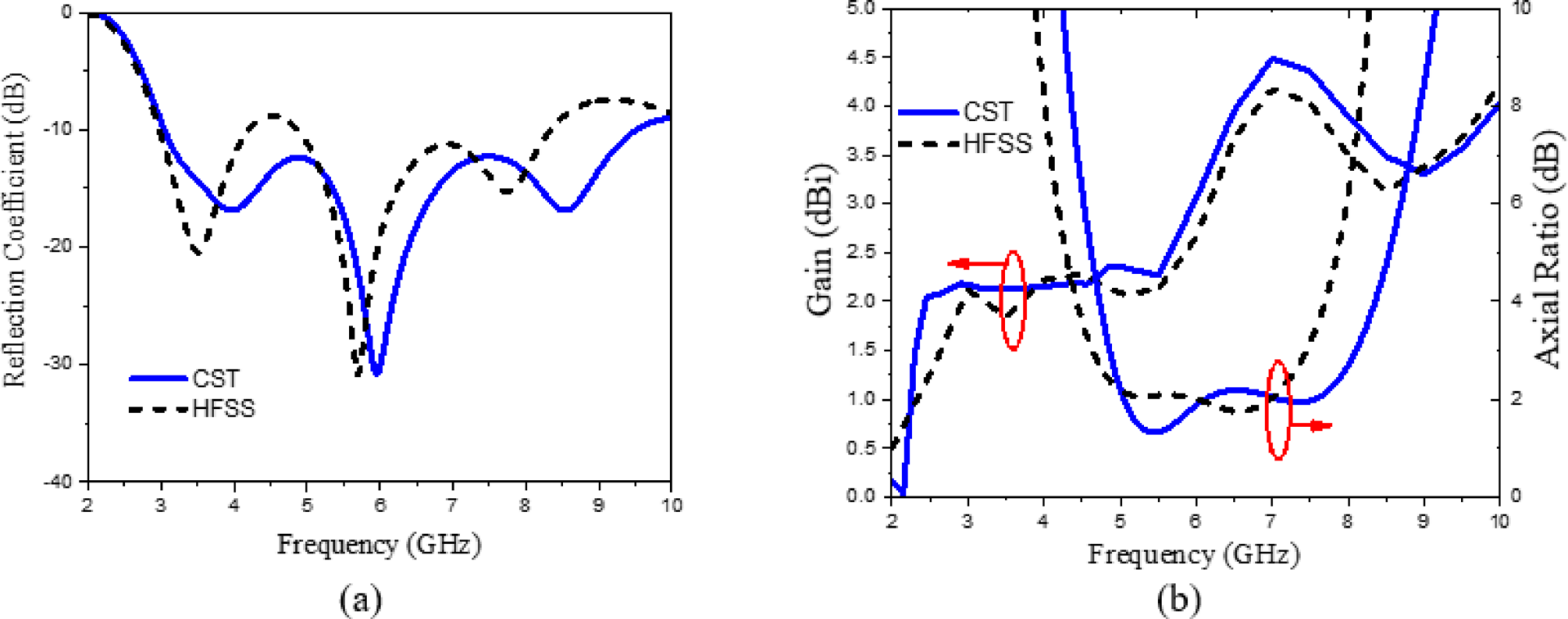
The radiating element: (**a**) Reflection coefficients versus frequency, (**b**) Gain, axial ratio versus frequency.

As illustrated in Fig. 5, the initial design operates within the frequency ranges of 3.52–5.26 GHz and 8.12–9.74 GHz. However, further enhancement of the bandwidth is necessary to meet the desired performance criteria. To enhance impedance matching, the second step involves modifying the monopole circular patch antenna by introducing a circular slot at the center of the rectangular ground plane, as illustrated in (Fig. 5a). As shown in Fig. 5b, this modification improves the S-parameter at 4 GHz from − 13.12 dB in the initial design to − 24.48 dB in the second step. In the final step, shown in Fig. 5a, a crescent-shaped radiating element is created by subtracting a circular shape along the x-axis from the top of the antenna, thereby improving impedance matching. Figure 5c–e indicate the gain and axial ratio variations versus frequency for each step. In general, the proposed antenna can accomplish a wide CP operation bandwidth by generating 2 adjacent CP bands formed by a semicircular patch and a circular slot. In fact, an original circular patch is not capable of generating CP waves. Consequently, it is subtracted from another eccentric circular shape centered at position (O) with a radius of (R_2_), as illustrated in (Fig. 4). This perturbation results in the generation of two orthogonal modes within the patch. Adjust the size and position of the eccentric circle, along with the feeding position, to ensure that these modes can be excited with equal magnitude and a phase difference of 90⁰, thereby enabling the realization of CP radiation. To expand the AR bandwidth, a circular slot is incorporated, functioning through a coupling mechanism with the patch to generate an additional CP band in the lower frequency range. The slot enhances both the impedance bandwidth and the axial ratio bandwidth by functioning as a radiator. The patch functions as a radiator to establish the upper CP band. The patch serves to achieve impedance matching and to feed the circular slot radiator located on the opposite side of the substrate, facilitating the creation of a lower CP band. The operational frequency of the slot is defined in relation to the TE11 mode of the circular slot, primarily influenced by R_2_, as indicated in Eq. (3)^51^. In this context, c represents the speed of light at 3 × 10^8^ m/s, $\:{\epsilon\:}_{r}$ denotes the dielectric constant of the substrate, and R_2_ indicates the radius of the circular slot etched in the ground plane. Figure 6 illustrates the polarization direction of the current at 5 GHz for various phase angles of the antenna, specifically at 0°, 90°, 180°, and 270°, facilitating the observation of CP performance. The phase angle of the current rotates in the clockwise direction, indicating that the current exhibits left-hand circular polarization (LHCP) in the broadside configuration. Additionally, the proposed antenna exhibits bi-directional radiation characteristics, with the current demonstrating right-hand circular polarization (RHCP) on the rear side. Figures 7 and 8 illustrate the current distribution at frequencies of 7 GHz and 8 GHz, respectively, for various phase angles of the antenna: 0°, 90°, 180°, and 270°. The proposed radiating element antenna demonstrates wideband characteristics, operating over a frequency range of 3.05 GHz to 9.51 GHz, achieving an impedance bandwidth of 102.8%, as seen in (Fig. 9a). The plot presented in (Fig. 9b) demonstrates that the antenna achieves a 3dB axial-ratio bandwidth of 50.6% within the frequency range of 4.83 to 8.11 GHz, and the simulated peak gain is 4.5 dBi.

3 $$\:{f}_{TE11}=\frac{1.84c}{4\pi\:{R}_{2}}\times\:\sqrt{\frac{1+{\epsilon\:}_{r}}{2{\epsilon\:}_{r}}}$$

**Fig. 10:**
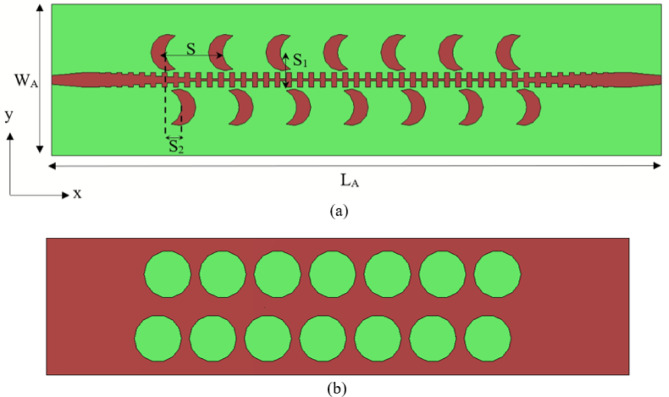
The proposed CP LWA Array geometry: (**a**) Top view, and (**b**) Bottom view.

The proposed CP leaky wave array antenna structure consists of radiating elements which is fed using STL on a grounded dielectric substrate, as shown in (Fig. 10). In the feeding part, the transmission line of the proposed antenna is the STL. The transition and tapered parts, denoted by L_1_ and L_2_ of the STL, are used to ensure 50 Ω impedance matching. In the transition region, the groove depth (d) is varied from 0.3 mm to 3 mm, as indicated in (Fig. 2b). Overall dimensions of the proposed array are given by L_A_ × W_A_ =297 × 70 mm^2^. The SPP waveguide exhibits a slow-wave mode with an SPP wavenumber exceeding the free-space wavenumber (k_SPP_ > k_O_). Therefore, it cannot radiate into free space. To obtain radiation, the inherent non-radiating mode can be transformed into a radiating mode by periodic perturbations along the SSPP structure. The periodic perturbations generate infinite number of space harmonics (Floquet modes), which can be written as^35^:

4 $$\:{\beta\:}_{n}={\beta\:}_{spp}+\:\frac{2n\pi\:}{p}$$

where $\:{{\upbeta\:}}_{\mathrm{s}\mathrm{p}\mathrm{p}}$ represents the wavenumber of the fundamental SSPP mode, $\:\mathrm{p}$ denotes the periodicity of the perturbations along the x-direction, and $\:\mathrm{n}$ corresponds to the harmonic order. In single-beam operations, the space harmonic of order $\:\mathrm{n}=-1\:$can be utilized, where it’s wavenumber is defined accordingly.

5 $$\:{\beta\:}_{-\:1}={\beta\:}_{spp}-\:\frac{2\pi\:}{p}$$

By adjusting the period $\:\mathrm{p}$, the wavenumber $\:{{\upbeta\:}}_{-\:1}$ can be either negative or positive, which allows the antenna’s radiated beam to be directed in either the forward or backward direction. The direction of the main beam can be expressed from $\:{\beta\:}_{-\:1}$ as described by wang et al.^52^.

6 $$\:\theta\:=\left\{\begin{array}{c}{{sin}}^{-1}\left({\beta\:}_{-\:1}/{k}_{o}\right)\\\:\pi\:\:-\:{{sin}}^{-1}\left({\beta\:}_{-\:1}/{k}_{o}\right)\end{array}\right.$$

**Fig. 11:**
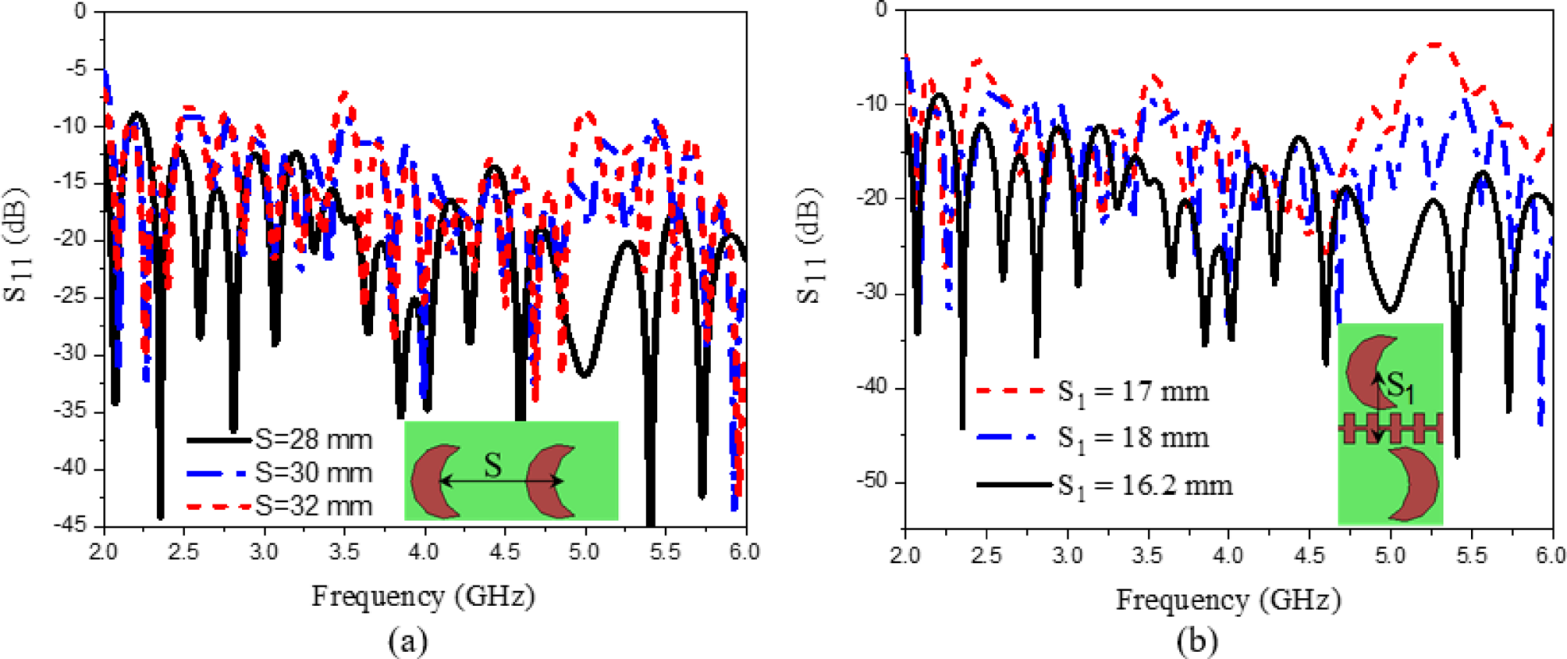
Reflection coefficient variation with frequency for CP LWA array at different values of (**a**) S, and (**b**) S_1_.

**Fig. 12:**
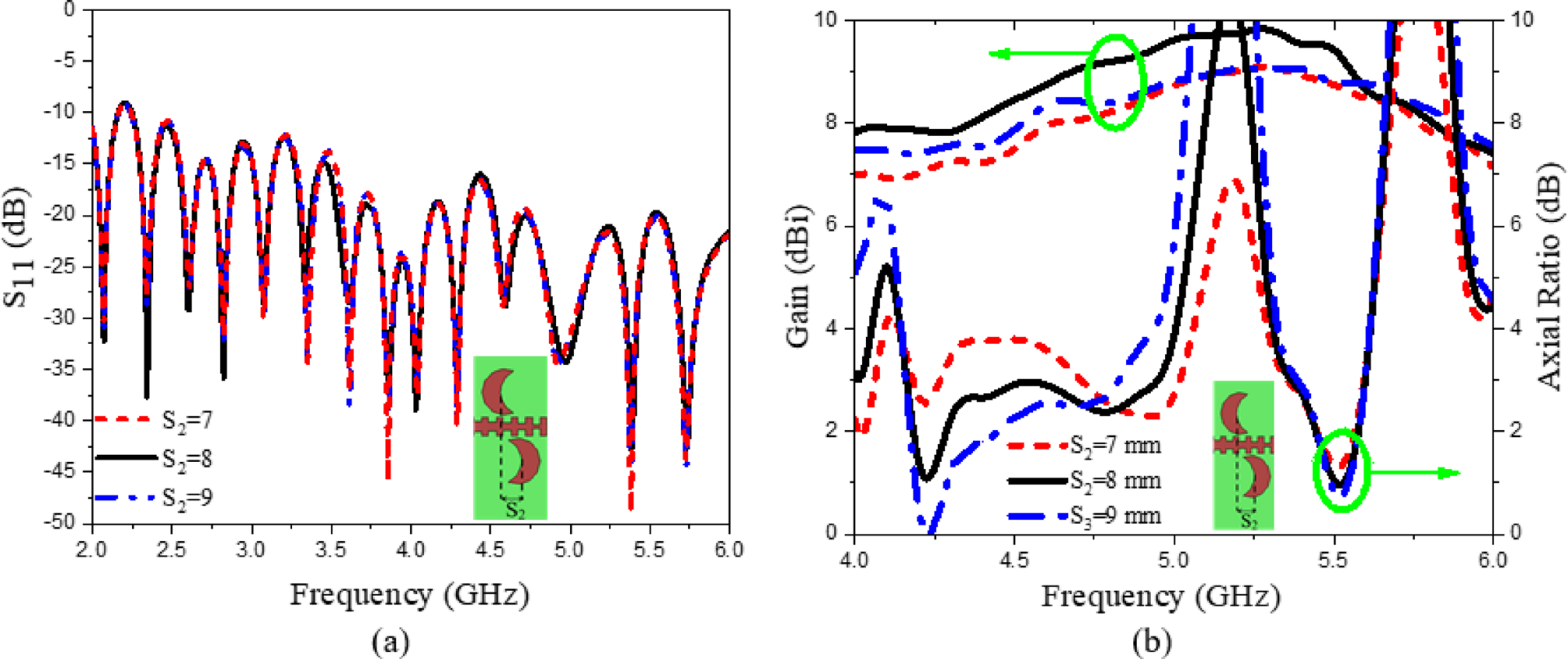
(**a**) Reflection coefficient variation versus frequency for CP LWA array at different separation values of S_2_, and (**b**) gain and axial ratio variation versus frequency for different S_2_ values.

Equation (6) characterizes the radiation beam in the upper half-space, whereas the subsequent equation defines the beam behavior in the lower half-space. In the proposed design, periodic perturbations are introduced through loading crescent patches positioned on both sides of the SPP waveguide, as illustrated in (Fig. 10a). When the patches are placed close to the SSPP waveguide, the fields couple to the patches through proximity feeding. The radiating part comprises a series of crescent-shaped metallic patches, which act as the radiating elements. In our design, the total number of elements is 14. While increasing the number of elements can enhance gain, it also expands the size of the array. The STL feeds these crescent-shaped patches through a coupled feeding mechanism^44^. The energy traveling along the SPP waveguide is progressively coupled to the patches, where it is transformed into radiated energy. The patches are placed at points where the magnetic field intensity is the highest to achieve optimal coupling^53^. The detailed design of the crescent-shaped patch is presented and discussed. The addition of each patch increases resistance due to radiation and introduces reactance, leading to slight changes in the field distribution and consequently affecting the SPP-guided wavelength (λg) of the STL^54^. Based on the dispersion curve in (Fig. 1b), at 5 GHz, β × *p* = 4/9π, the guided wavelength of the STL is λg = 2π/β = 24 mm. To achieve radiation at 5 GHz, the period of the loaded radiating element is λg = 24 mm^35^. The separation between adjacent patches (S), the positional distance (S_1_) of the loaded crescent patches along the STL, and the offset distance (S_2_) between the lower and upper crescent patches along the positive z-axis are key design parameters. Parametric analysis was conducted on the three key distances (S, S_1_, and S_2_), as illustrated in (Figs. 11 and 12). The results indicate that S and S_1_ affect the impedance matching characteristics, while variations in S_2_ primarily affect the gain and axial ratio but have no impact on the impedance matching. S_2_ is equal to 8 mm for achieving CP. According to 5 GHz = 24 mm. Here in the design, S_2_ is optimized to get CP at different frequencies of 4, 4.5, 5, and 5.5 GHz. The optimal separation between the patches is set to S = 28 mm, the positional distance is S_1_ = 16.2 mm, and the offset distance between the lower and upper patches is S_2_ = 8 mm. To enhance the CP radiation of the proposed design, circular-shaped slots are incorporated into the ground plane, as described in detail in the radiating element design. The curves shown in Figs. 11 and 12 depict the relationship between reflection coefficients and frequency, as well as the axial Ratio and gain vs. frequency. This study focuses on tuning three key parameters: the separation between adjacent patches (S), the loaded circular patches positional distance (S_1_) along the STL, and the offset distance between the lower and upper patches (S_2_). Specifically, the separation S varies between 28 mm and 32 mm, the positional distance S_1_ ranges from 16.2 mm to 18 mm, and the separation S_2_ spans from 7 mm to 9 mm. The circular polarization can be further enhanced by tuning the positional parameter S_1_ and the horizontal separation parameter S_2_.

**Fig. 13:**
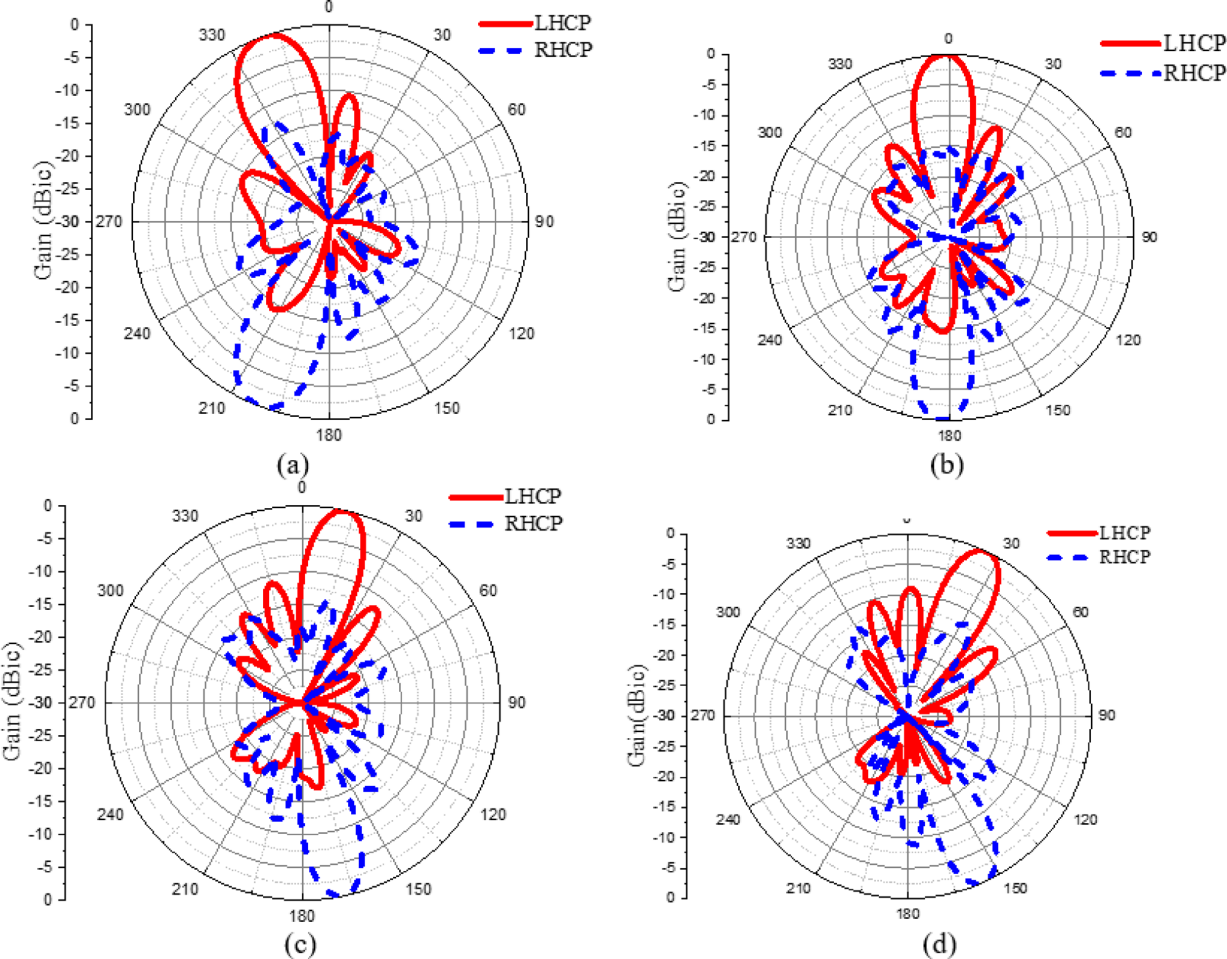
Normalized LHCP and RHCP radiation patterns of CP LWA array at different frequencies; (**a**) 4 GHz, (**b**) 4.5 GHz, (**c**) 5 GHz, (**d**) 5.5 GHz.

**Fig. 14:**
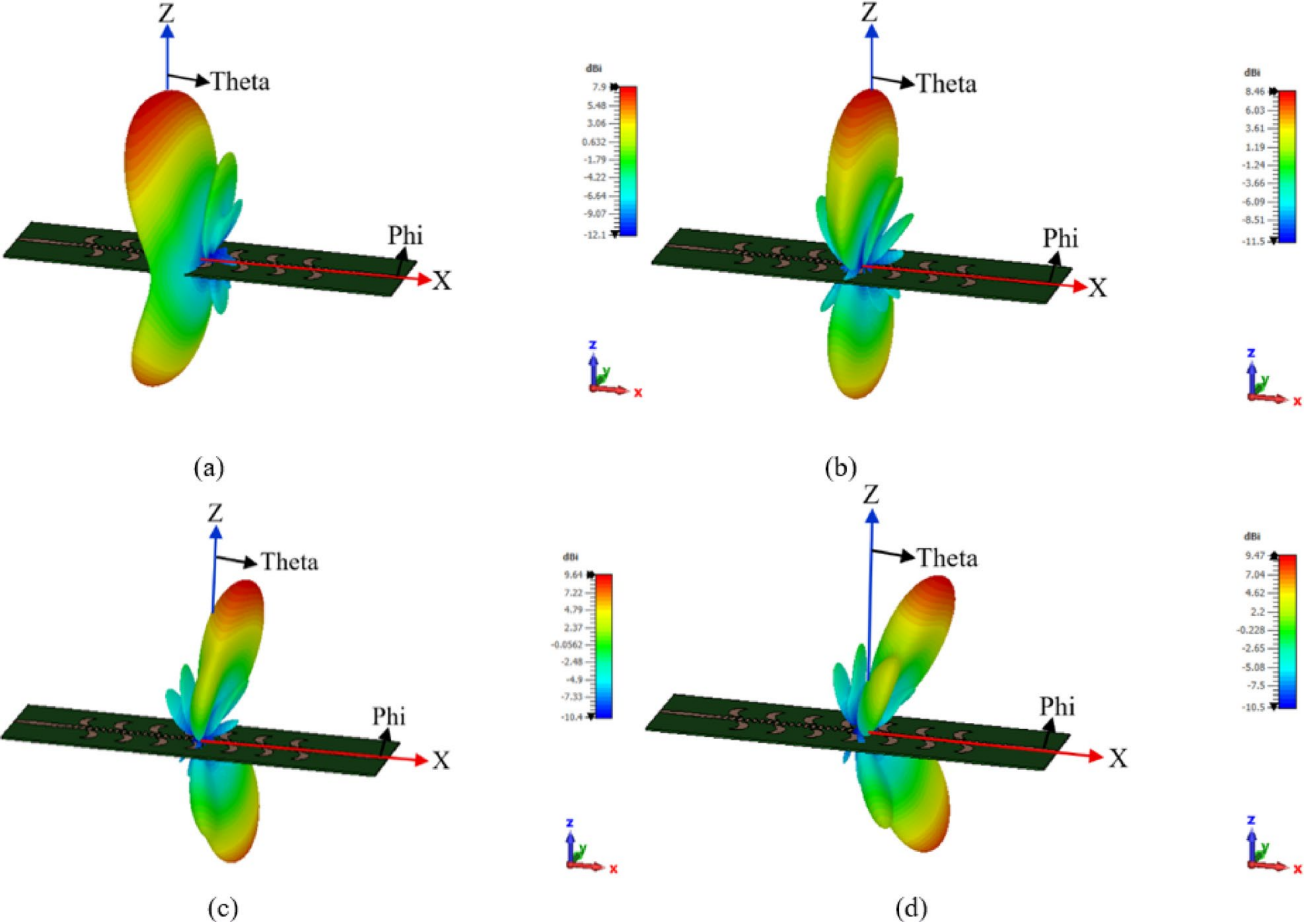
3-D radiation pattern of CP LWA array at different frequencies; (**a**) 4 GHz, (**b**) 4.5 GHz, (**c**) 5 GHz, (**d**) 5.5 GHz.

The LHCP and the RHCP radiation patterns of the CP LWA array is shown in (Fig. 13). It can be noticed that the proposed CP LWA array has slots in the ground plane, consequently the array radiates in both directions. If in the upper side is left hand then the backward side is right hand. The 3-D radiation pattern of the proposed CP LWA array is shown in (Fig. 14). The beam scanning range of the CP LWA is extended to *50°*, spanning from − *21°* to + *29°*, with the gain varying from 7.9 dBi to 9.64 dBi, as illustrated in (Fig. 14).

**Fig. 15:**
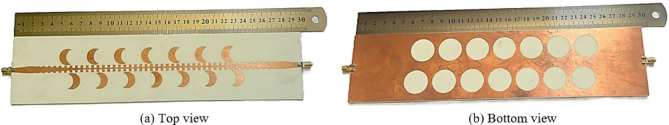
Fabricated photo of CP LWA array. (**a**) Top view, (**b**) Bottom view.

**Fig. 16:**
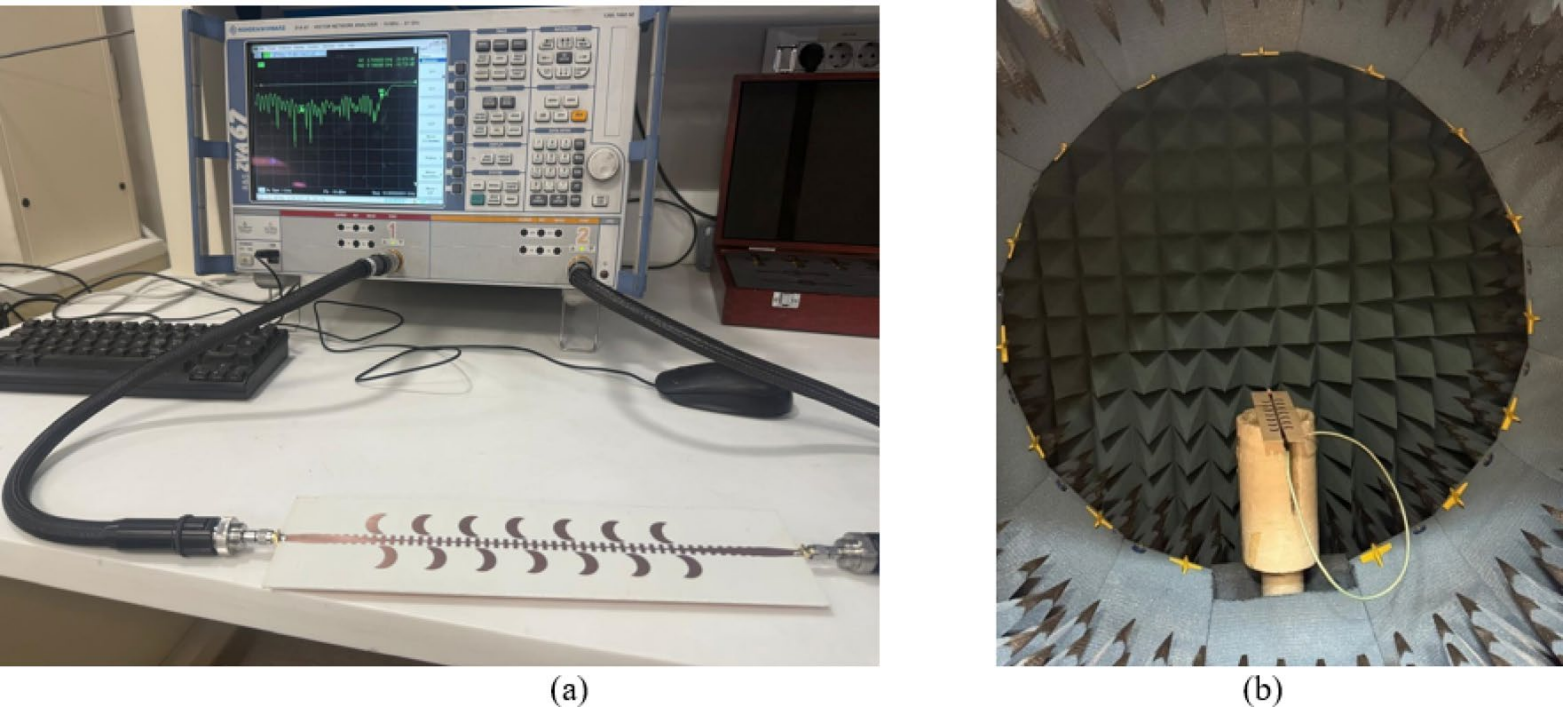
The proposed CP-LWA during the measurements of (**a**) scattering parameters, and (**b**) Far-field measurements.

**Fig. 17:**
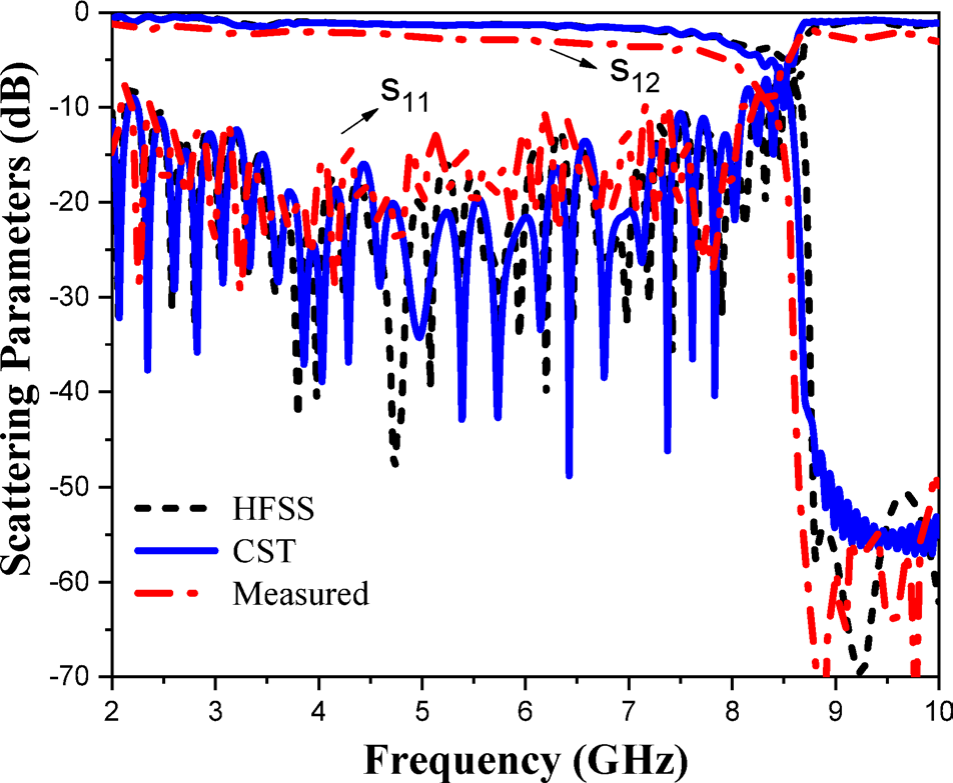
Simulated and measured scattering parameters of the CP LWA array.

**Fig. 18:**
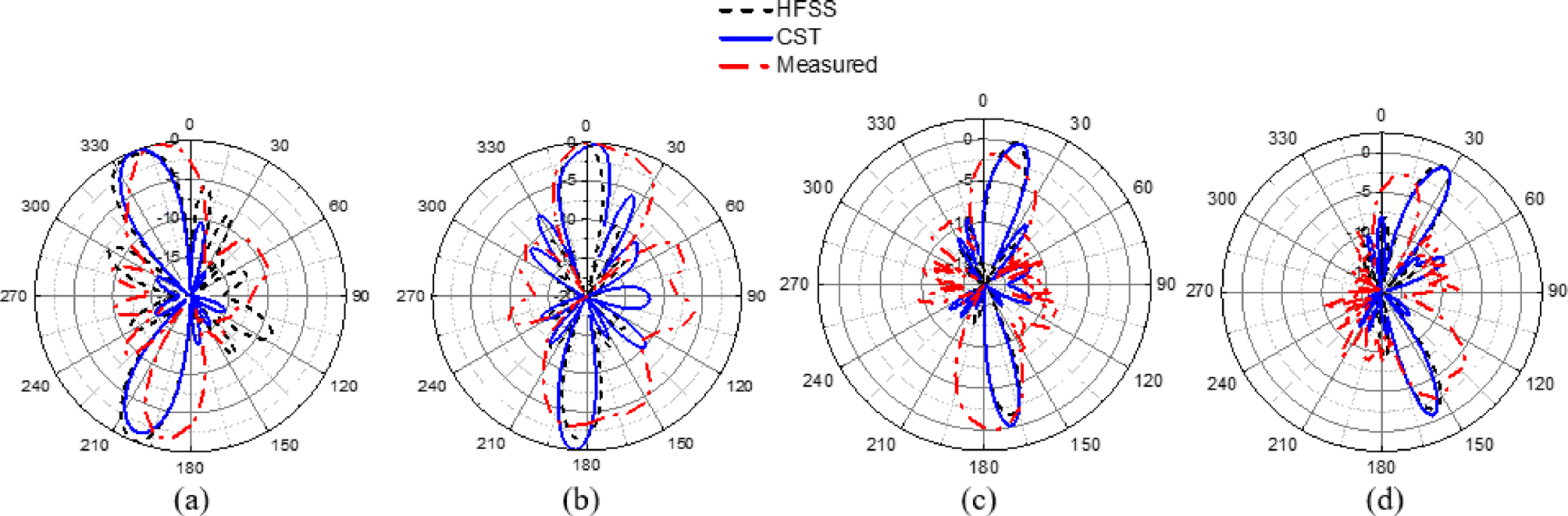
Simulated and measured 2D radiation pattern at frequencies: (**a**) 4 GHz, (**b**) 4.5 GHz, (**c**) 5 GHz, (**d**) 5.5 GHz.

**Fig. 19:**
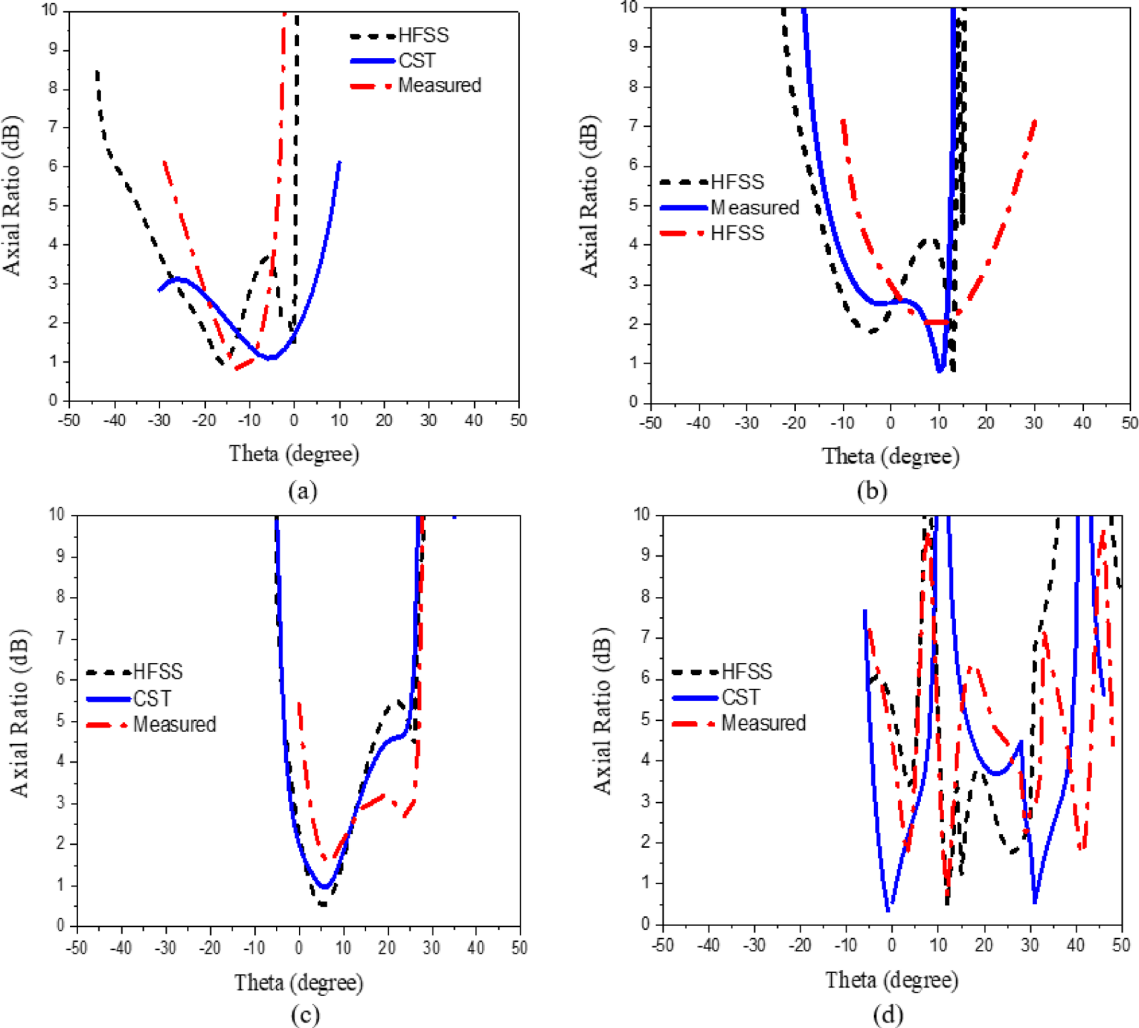
Simulated and measured axial ratios versus theta at different frequencies: (**a**) 4 GHz, (**b**) 4.5 GHz, (**c**) 5 GHz, (**d**) 5.5 GHz.

**Fig. 20:**
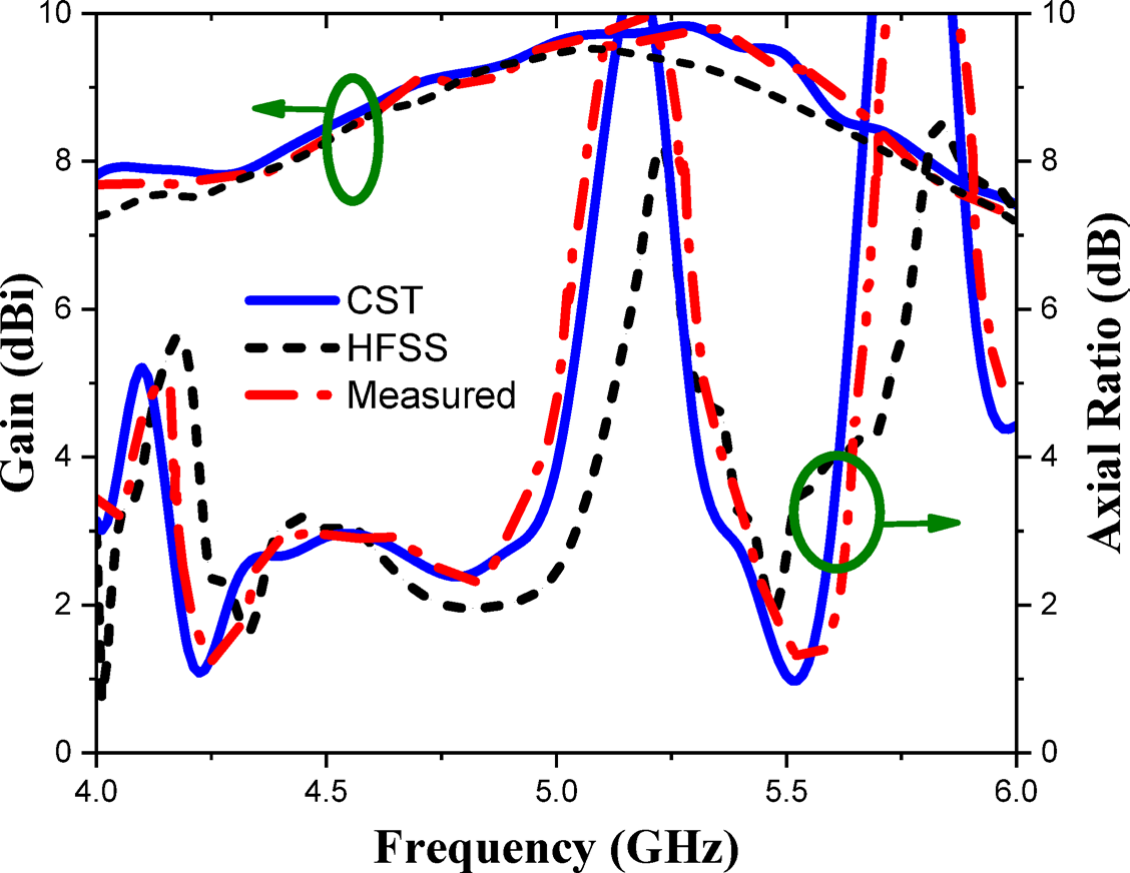
Simulated and measured gain and axial ratio versus frequency of CP LWA array.

The CP LWA array was fabricated and measured to validate the preceding analysis and measure its performance. Fabricated photo of the CP LWA array is shown in (Fig. 15). The top view shows the implementation of crescent-shaped patches on both sides of the STL, while the bottom view depicts the circular slot embedded in the ground plane. The reflection and transmission coefficients of the CP LWA array are measured using an Agilent ZVA-67 (Rohde & Schwarz) vector network analyzer (VNA), as shown in (Fig. 16a). There is good agreement between the simulated, measured reflection and transmission coefficients as can be seen in (Fig. 17). It is observed that the reflection coefficient (S_11_) remains below − 10 dB over the 2–8 GHz frequency range, indicating effective impedance and momentum matching. Additionally, the transmission coefficient (S_12_) remains satisfactory across the entire operating bandwidth, indicating that the excitation of the patch array via the SSPP waveguide has been effectively achieved. The radiation characteristics of the CP LWA array are measured in the anechoic chamber (Star Lab.-007-A-0019) using a far-field measurement setup over the frequency range of 2 GHz to 8 GHz, as shown in (Fig. 16b). Figure 18 presents the 2D simulated and measured radiation patterns at 4, 4.5, 5, and 5.5 GHz. Figure 19 presents simulated and measured axial ratios variations versus theta at various frequencies. Beam directions and axial ratios values are listed in (Table 1). Table 2, summarizing gain, SLL, and beamwidth at different frequencies of 4, 4.5, 5, and 5.5 GHz, is presented to fully evaluate the practical performance of the antenna across this range of frequency. Figure 20 shows the simulated and measured gain and axial ratio versus frequency of the proposed CP LWA array.

**Table 1:** Simulated and measured beam directions, and axial ratios at different frequencies.

Frequency (GHz)	Beam direction (degree)	Axial ratio ((dB) simulation CST	Axial RATIO ((dB) simulation HFSS	Axial ratio ((dB) measured
4	-21	3.2	2	3.2
4.5	2	2.5	2.9	2.6
5	13	2.9	2.8	2.7
5.5	29	2.6	2.2	2

**Table 2:** Simulated and measured gains, side lobe levels, and beamwidths at different frequencies.

Frequency (GHz)	Gain (dBi) simulation CST	Gain (dBi) simulation HFSS	Side lobe level (dB) simulation CST	Side lobe level (dB) simulation HFSS	Side lobe level (dB) measured	Beamwidth (degree) simulation CST	Beamwidth (degree) simulation HFSS	Beamwidth (degree) measured
4	7.9	7.8	-10.132	-11.305	-9.425	21.4	19.1	28.111
4.5	8.46	8.36	-9.629	-9.882	-6.263	16.4	17.954	16.724
5	9.64	9.54	-11.35	-11.371	-9.387	14.2	14.98	13.953
5.5	9.47	9.37	-11.605	-11.615	-5.156	14.9	14.364	12.517

## Design of the rectifier circuit

The rectifier is a crucial component within the rectenna system, and is used to convert ambient RF energy into a DC power. The rectifier circuit consists of a diode, a DC pass filter, a load resistor, and an impedance matching network (IMN). The IMN optimally matches the receiving antenna and the rectifier circuit at the desired operating frequency, thereby maximizing power conversion efficiency and reducing transmission loss. A diode is an essential component in a rectifier circuit, which converts RF energy into direct current (DC). A diode exhibiting a low turn-on voltage (Vth) is a crucial parameter for efficient operation with low-power RF signals. A Schottky diode is ideal for rectifier design due to its low turn-on voltage and effectiveness at radio frequencies. A high-efficiency rectifying circuit is crucial for designing rectennas, enabling an effective energy harvesting system. Various rectifier shapes were analyzed for converting received RF power into DC, such as series and shunt diode rectifiers, full-wave bridge rectifiers, voltage doubler rectifiers, and Greinacher rectifier circuits^15^. The conventional single-diode series/shunt topology has limited energy conversion efficiency^15^. In contrast, full-wave bridges and Greinacher rectifiers use additional diodes, which leads to increased losses that negatively impact the overall performance of the rectifiers. Therefore, a series-pair rectifier configuration (voltage doubler) is used to achieve high power conversion efficiency (PCE), which is essential for powering low-power electronic devices^15^.

**Fig. 21:**
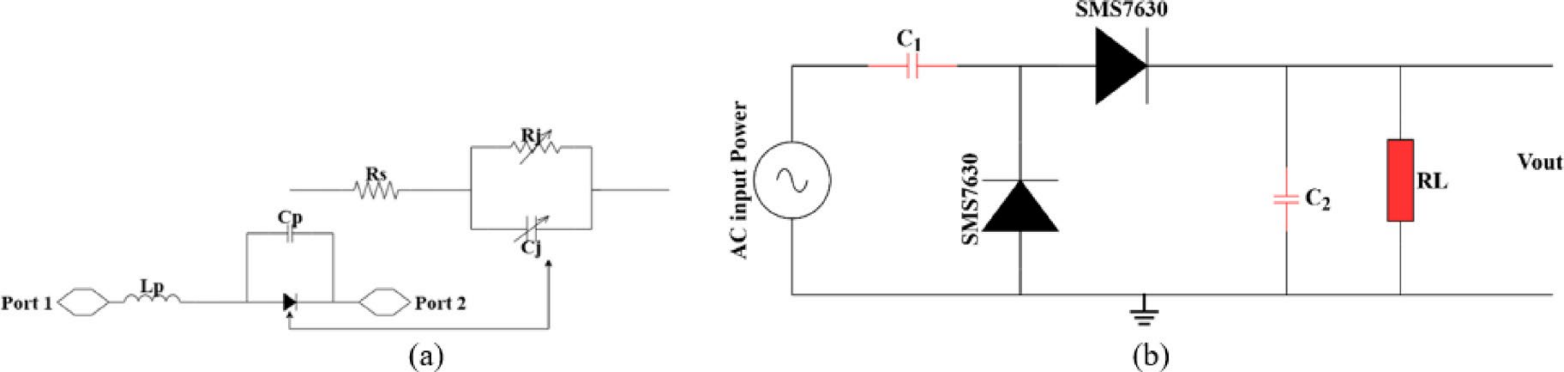
Proposed rectifier circuit; (**a**) Diode model and equivalent circuit (**b**) Schematic view.

**Fig. 22:**
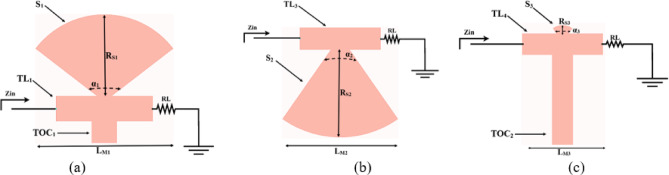
The sections of the wideband impedance matching network: (**a**) Sect. 1, (**b**) Sect. 2, and (**c**) Sect. 3.

**Fig. 23:**
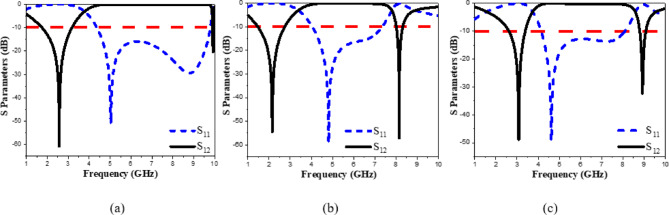
S parameters of the sections of the impedance matching network: (**a**) Sect. 1, (**b**) Sect. 2, and (**c**) Sect. 3.

**Fig. 24:**
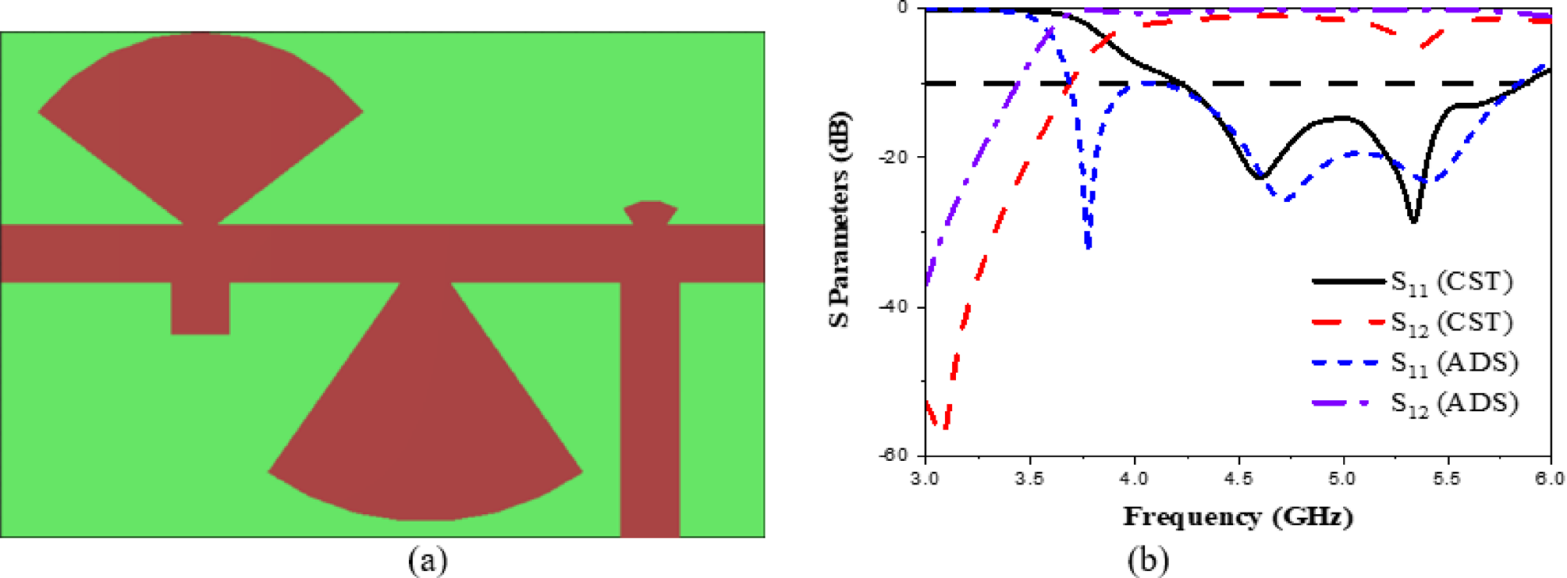
The layout and simulated s parameters of the wideband impedance matching network.

**Fig. 25:**
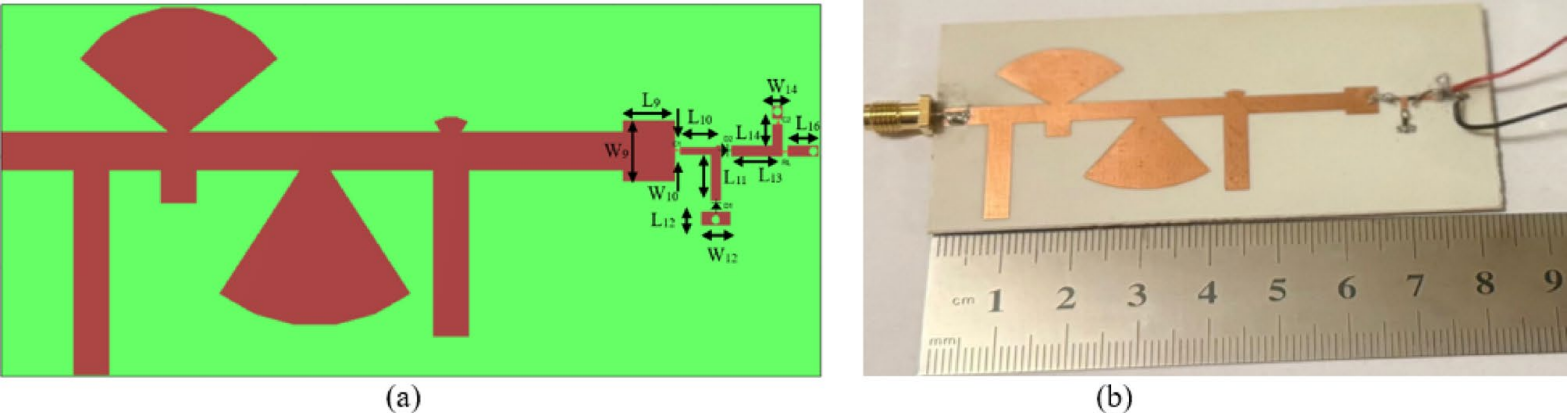
The layout and fabricated photo of wideband rectifier.

**Fig. 26:**
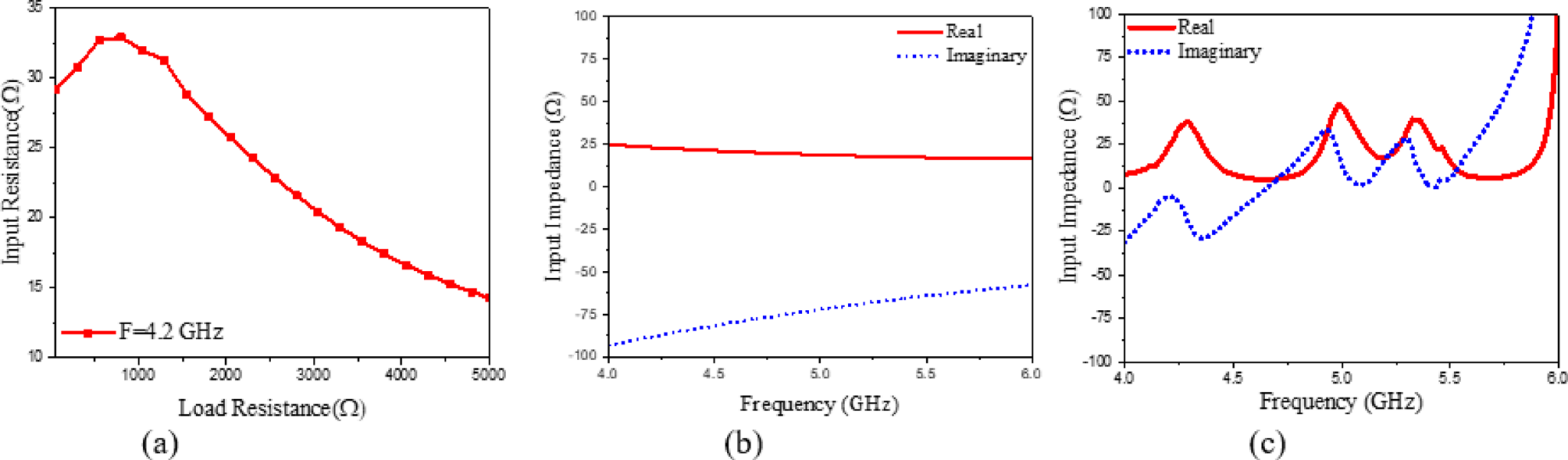
(**a**) Input resistance versus load resistance at 4.2 GHz at 0dBm input power, (**b**,**c**) Input impedance versus frequency of the rectifier before applying WRCN, and after applying WRCN, respectively.

**Fig. 27:**
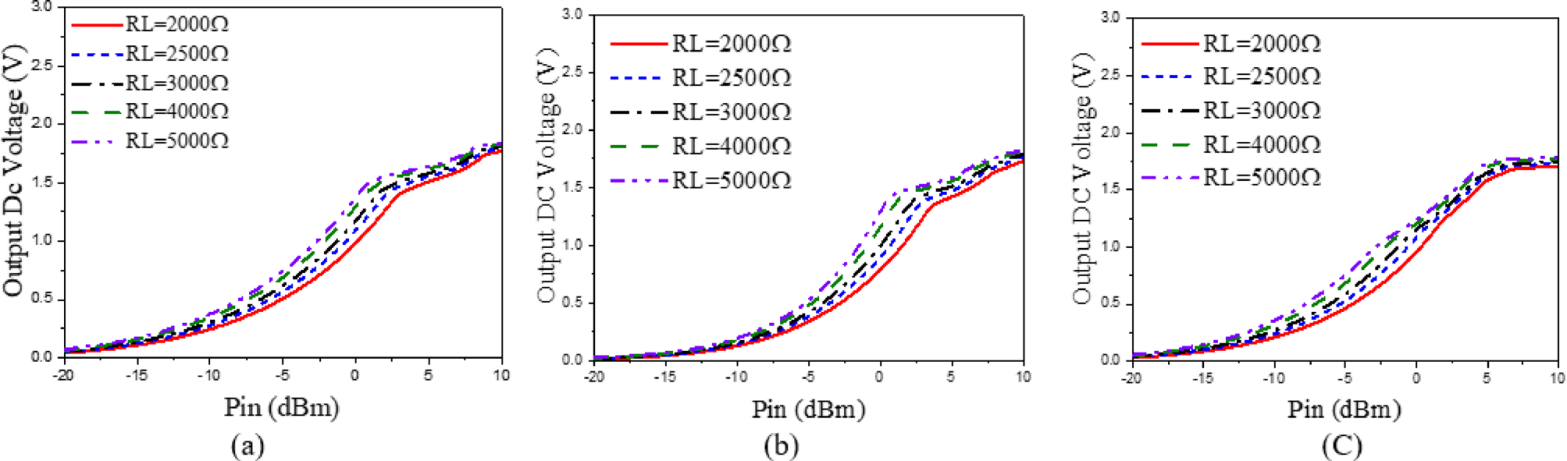
Simulated output DC Voltage versus different power levels at different resistive loads; (**a**) 4.2 GHz, (**b**) 4.4 GHz, and (**c**) 5 GHz.

**Fig. 28:**
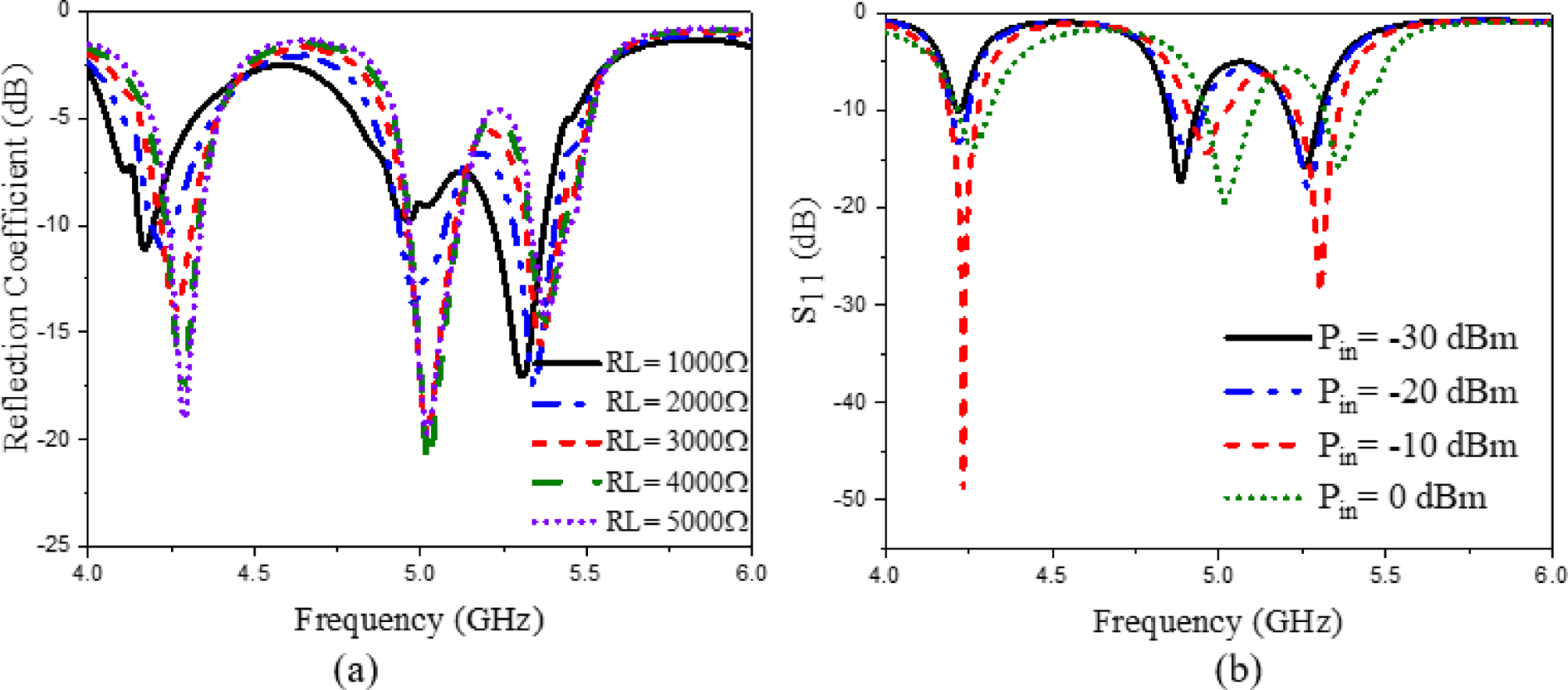
(**a**) The effect of load (RL) variation on the rectifier reflection coefficient at Pin = 0dBm, (**b**) the effect of input power (Pin) variation on the rectifier reflection coefficient at RL = 2500 $\:{\Omega\:}$.

**Fig. 29:**
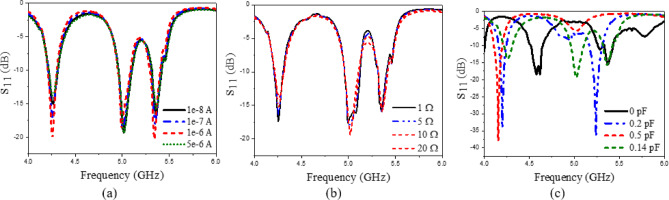
The rectifier reflection coefficient versus frequency at RL = 2500 Ω, Pin = 0dBm for different diode parameters of (**a**) Saturation current Is, (**b**) Series resistance Rs, and (**c**) Nonlinear junction capacitance Cjo.

The presented rectifier is based on voltage doubler circuit and uses a Schottky diode, specifically the SMS7630 model from Skyworks with zero-biasing. It has a low forward junction capacitance (0.14 pF), low series resistance (20 Ω), low turn-on voltage, and high reverse saturation current (5 µA)^55^. The equivalent circuit of the selected diode is shown in (Fig. 21a), with parasitic components Lp = 0.7 nH and Cp = 0.07 pF. The rectifier circuit is presented in (Fig. 21b). The design employs a voltage doubler topology comprising a series pair of SMS7630 Schottky diodes, a DC blocking capacitor, a DC pass filter capacitor, and a resistive load. A transmission line based on a wideband resistance compression network^56^ is utilized. It includes three impedance transformation networks that use radial lines and open-circuited stubs. The wideband matching network (WMN) is shown in (Fig. 24a). It consists of three sections, as illustrated in (Fig. 22). Section 1 contains the radial stub S_1_, the microstrip line TL_1_, and the opened stub TOS_1_. Sections 2 and 3 are made up of TL_3_ and TL_4_ combined with S_2_ and S_3_, combined with TOC_2_, respectively. The radial stub is used in Sects. 1, 2, and 3 because of its wide stopband characteristics. The radius of S_1_, S_2_, and S_3_ corresponds to a specific value ($\:{\lambda\:}_{g}$/4), where is the guided wavelength at the operating frequency. The lengths of TOC_1_ and TOC_2_ are almost equivalent to this value ($\:{\lambda\:}_{g}$/4). Adjusting the radius of the three radial stubs and the length of the two open-circuited stubs allows the three sections to operate over different frequency bands. The optimized dimensions of each section employed in the design WMN are: L_M1_ = 13.5 mm, R_S1_ = 10.85 mm, α_1_ = 105^⁰^, L_M2_ = 13 mm, R_S2_ = 13.6 mm, α_2_ = 70^⁰^, L_M3_ = 13.5 mm, R_S3_ = 1.1 mm, and α_3_ = 68^⁰^. Figure 23 shows the simulated S-parameters for each section. Figure 24b demonstrates that the simulated scattering parameters of the wideband matching network by using Advanced Design System (ADS) and CST remain consistently below − 10 dB across the 4.1–5.5 GHz frequency range, indicating effective impedance matching and stable wideband performance. The layout and the fabricated photo of the proposed rectifier are illustrated in (Fig. 25a, b), respectively. The SMS7630 Schottky diode in a Skyworks package is employed in a voltage doubler topology, with the design implemented on an RO4003C substrate of 1.52 mm thickness. The matching network consists of the previously discussed WMN, a short-circuit stub in series with the voltage doubler’s shunt diode, and three series transmission line segments. This compression network operates to confine the input resistance within a narrow range, while TL_1_ and TOC_1_ are designed to match the input impedance to 50 Ω. The resistive load is 2500 Ω. The total dimensions of the rectifier are 79.5 × 33.3 × 1.52 mm³. The optimized transmission line dimensions (in mm) employed in this design are summarized in (Table 3). The advantage of proposed matching network in the manuscript over using one section is that it compresses the input impedance^56^ from 15 to about 30 ohms which leads to wider matching and stable bandwidth with the load variation from 50 up to 5000 ohms at 0dBm and 4.2 GHz as indicated in (Fig. 26a). The input impedance plots versus frequency of the rectifier before and after applying a wideband resistance compression network, are shown in Fig. 26b, c, respectively. Figure 27 presents the simulated output DC voltage at various power levels, resistive loads, and frequencies. The reflection coefficient variation versus frequency for different values of load (RL) at Pin = 0dBm is shown in (Fig. 28a), and the effect of input power (Pin) variation on the rectifier reflection coefficient at RL = 2500 Ω is shown in (Fig. 28b). Furthermore, the rectifier sensitivity to load, input power variations is studied according to the reflection coefficient versus frequency, and the results are shown in (Fig. 29). These results state clearly that the proposed WRCN shows good resistance compression performance in operating frequency bands. Furthermore, the nonlinearity of diode is studied according to different parameters of Saturation Current Is, Series Resistance Rs, and Nonlinear junction capacitance Cjo. Figure 29a–c present the rectifier reflection coefficient versus frequency at RL = 2500 Ω, Pin = 0dBm for different diode parameters of Is, Rs, and Cjo, respectively. The reflection coefficient of the rectifying diode demonstrates a powerful nonlinear correlation with frequency, significantly affected by the intrinsic parameters of the diode. Changes in the saturation current (Is) affect the diode’s turn-on characteristics and dynamic resistance. This results in frequency-dependent alterations in the input impedance and, consequently, the reflection coefficient. An increase in (Is) typically leads to a reduction in effective junction resistance, enhancing impedance matching at lower frequencies and modifying the resonance behavior throughout the operating band. The series resistance (Rs) leads to additional ohmic losses that become more significant at higher frequencies, resulting in increased reflection and reduced power transfer efficiency, particularly near resonance. Additionally, the nonlinear junction capacitance (Cjo) significantly affects the reactive component of diode impedance. This leads to variations in the reflection coefficient that depend on frequency, altering the bandwidth and matching stability. As Cjo varies with voltage and frequency, it causes dispersion effects that can change the impedance response at different excitation levels. The combined nonlinear effects of Is, Rs, and Cjo significantly influence the diode’s reflection coefficient across frequencies, highlighting the necessity for precise diode modeling to effectively design wideband rectennas.

**Fig. 30:**
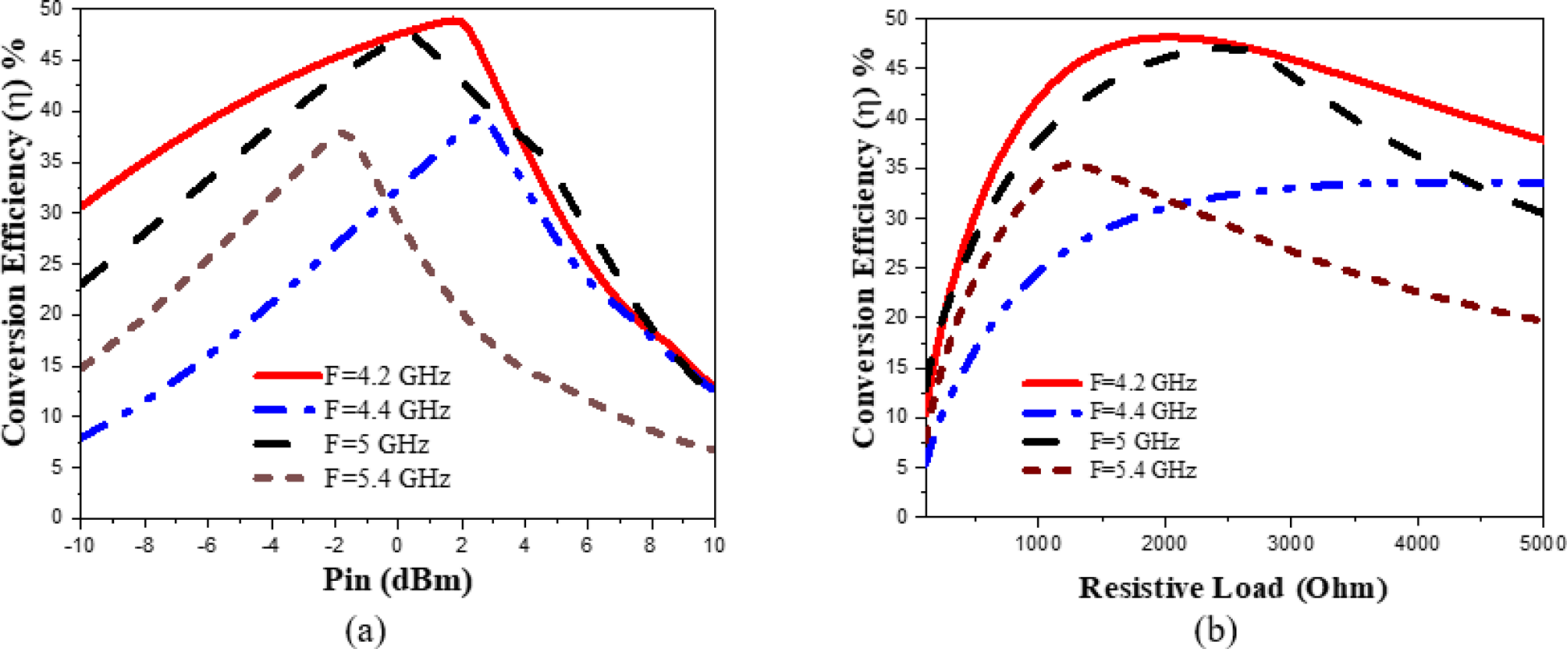
Simulated performance; (**a**) PCE vs. Pin at different frequencies, and (**b**) PCE vs. resistive load at different frequencies, with 0 dBm input power.

**Fig. 31:**
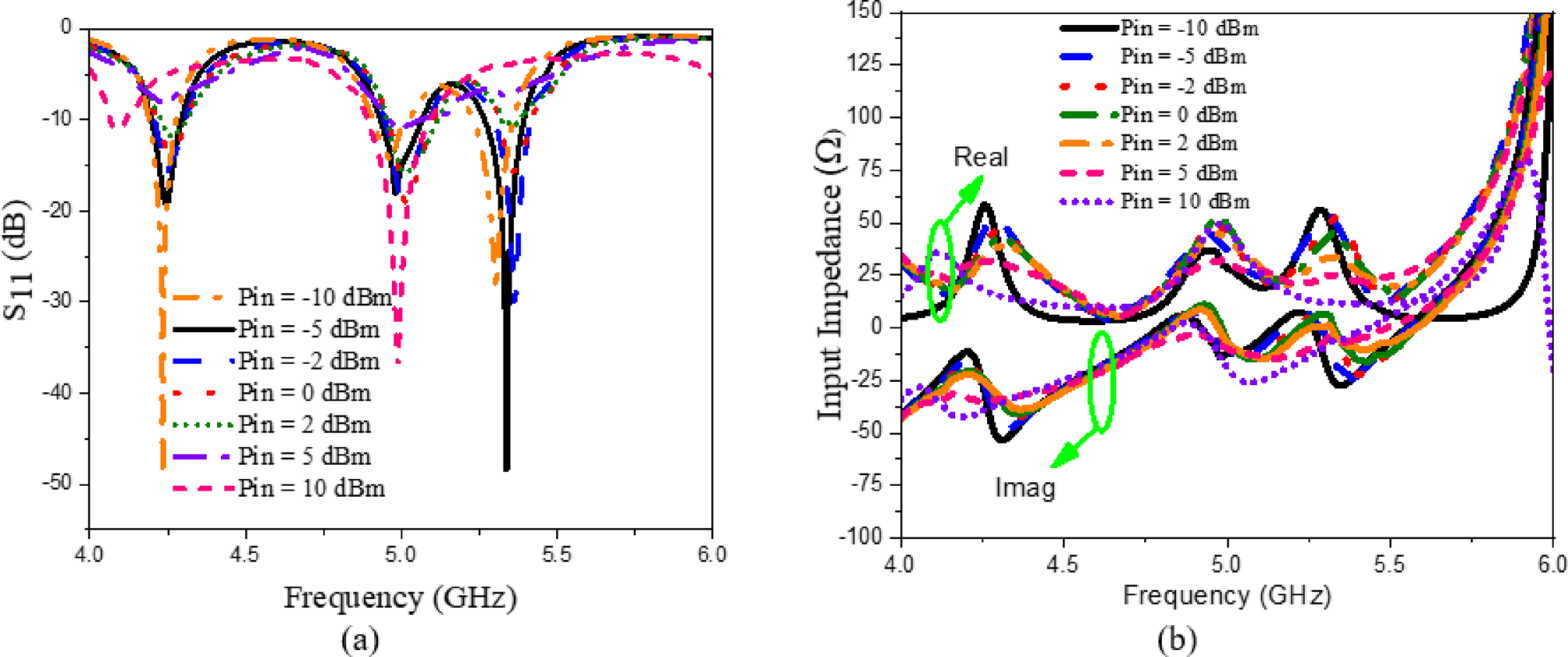
Rectifier measured performance at different P_in_ levels: (**a**) S_11_ versus frequency, and (**b**) Input impedance versus frequency.

The rectifier was simulated using ADS with harmonic balance and large-signal S-parameter analysis. The PCE within the specified band was evaluated across varying load values and input power levels. The rectifier’s performance at a given input power level depends on the load value, which can be obtained using Eq. (7)^57^. The maximum output voltage (V_outMax_) is constrained to half the breakdown voltage. The maximum output power (P_outMax_) is proportional to the square of the diode breakdown voltage (V_br_) and inversely proportional to the rectifier resistive load (R_L_). The PCE of the rectifier can be determined through Eq. (8). This expression relates the ratio of the rectifier’s output direct current (DC) power to the input radio frequency (RF) power, thereby providing a quantitative measure of how effectively the circuit converts incident RF energy into usable DC power. The optimization procedure for selecting the optimal load value involves evaluating rectifier performance across a range of input power levels and load values. Figure 30a illustrates the PCE as a function of input power across various frequencies within the 4–5.4 GHz range. The maximum power conversion efficiency (PCE) achieved was 48.92% at an input power of 1.75 dBm and a frequency of 4.2 GHz. Figure 30b shows the PCE versus resistive load at different frequencies. It can be seen that for each frequency value, there is an optimum load value to achieve maximum efficiency. At 4.2 GHz, the maximum efficiency is achieved at a 2000 Ω load with 0 dBm input power. Rectifier measured matching performance at different P_in_ levels is presented in (Fig. 31).

## Integration of CP LWA array with rectifier

**Fig. 32:**
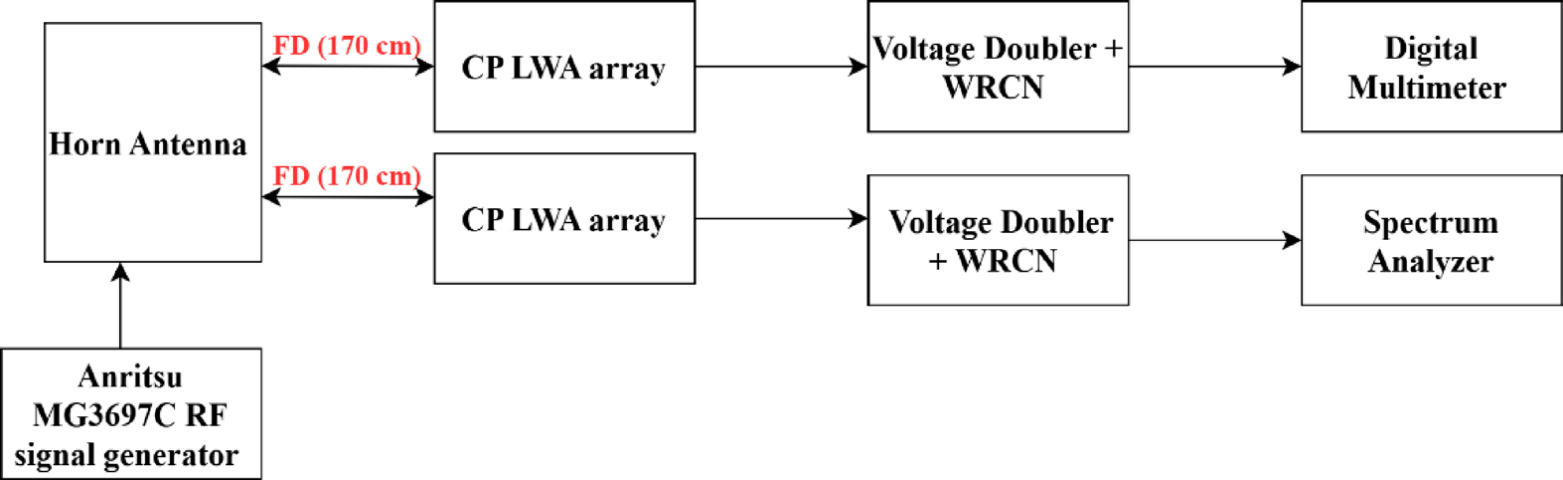
The block diagram of the proposed experimental setup for measuring the rectenna system.

**Fig. 33:**
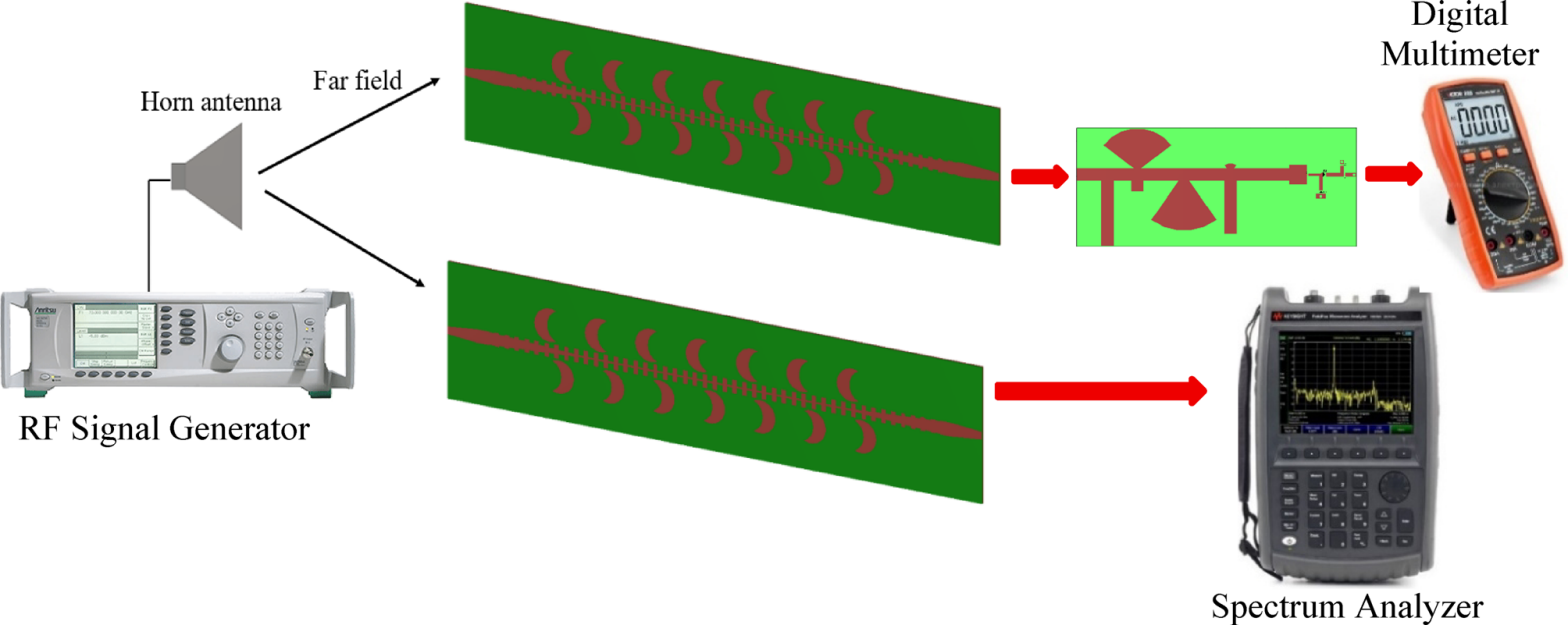
The proposed experimental setup for measuring the rectenna system.

**Fig. 34:**
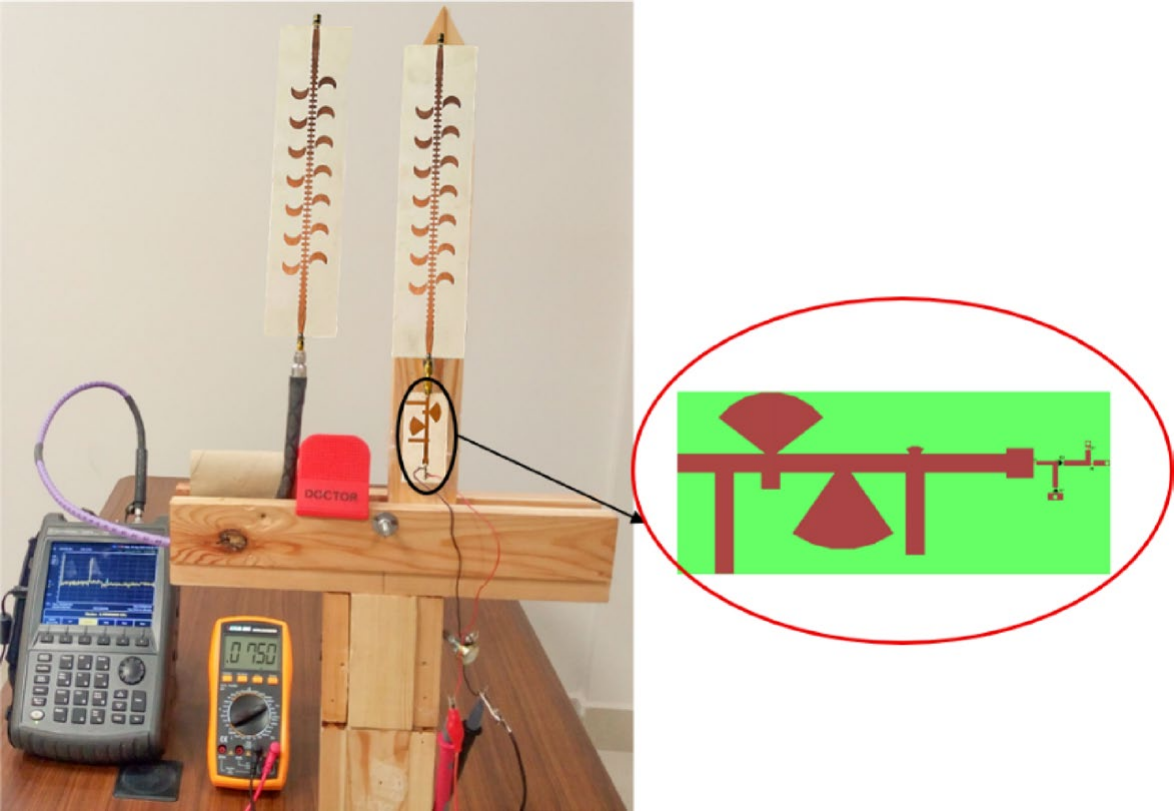
Photo taken during the measurement process indicates DC output volt at received power by the antenna of −3.7 dBm and F = 4.2 GHz.

**Fig. 35:**
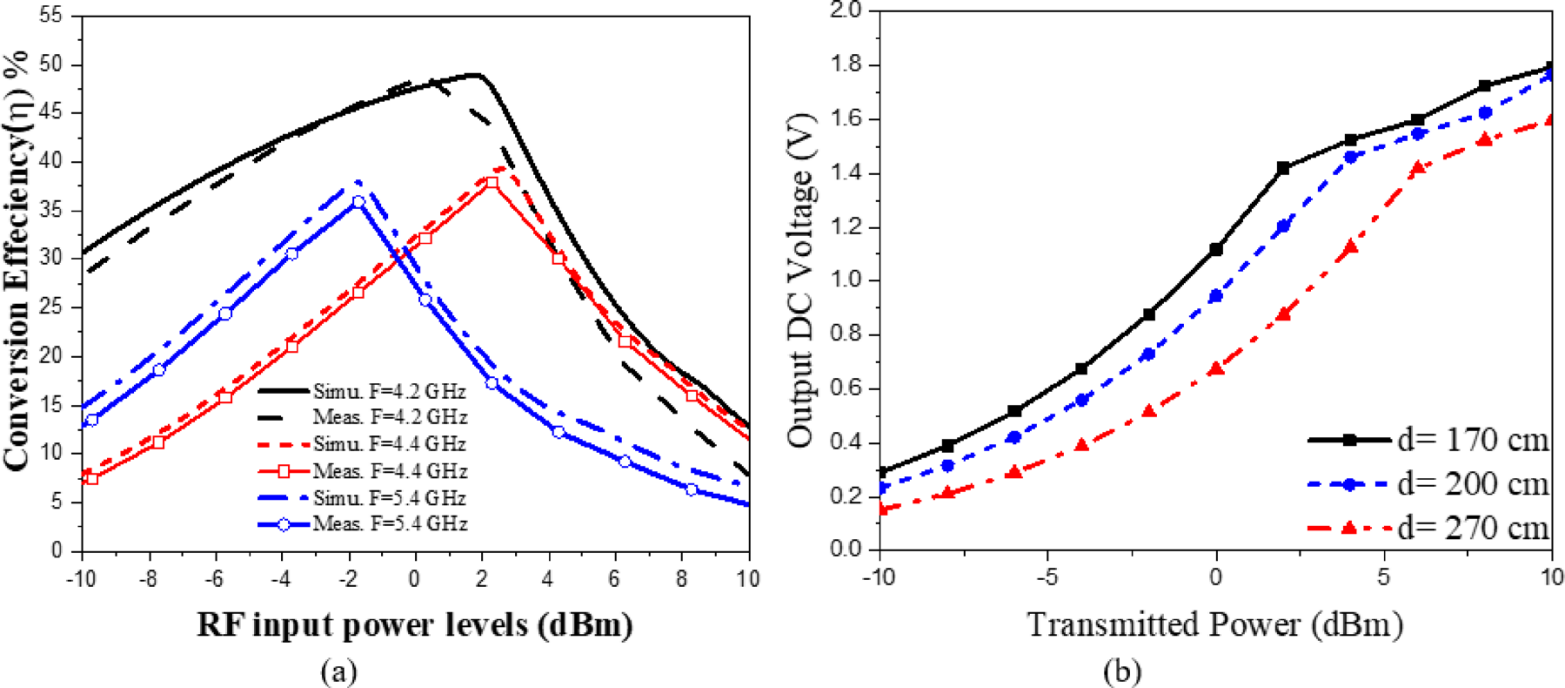
(**a**) The measured efficiency variation versus RF received power, (**b**) Measured DC output Voltage versus transmitted power at different distance between the transmitting horn and the proposed rectenna.

The proposed CP LWA antenna is used in receiving mode to harvest the RF waves at the frequency band of 4 to 5.5 GHz. The horn antenna is placed at a fixed distance from the rectenna, 170 cm, to provide a controlled RF signal across a broad frequency spectrum (4–6 GHz). The distance between rectenna and horn antenna is 170 cm (the far field distance of transmitted horn antenna at 4.2 GHz. The experimental methodology is as follows: Fig. 32 displays the block diagram of the measurement setup for testing the proposed CP rectenna system. The rectenna is integrating by connecting a wideband CP LWA array to the multiband rectifier through a coaxial adapter. To evaluate the rectenna’s performance, hardware measurements were conducted to determine its PCE. The RF signal generator was used to feed the LB-7180-NF Broadband Horn antenna^58^ functioning as the transmitter, while two identical receiving antennas were positioned at a fixed distance from the horn antenna. The first antenna was connected to a spectrum analyzer to record the received RF power. At the same time, the second antenna was integrated with a designed rectifier circuit. Different transmit power levels were utilized to feed the horn antenna. The received RF power at the proposed CP LWA antenna was measured at each level using a spectrum analyzer, and the corresponding output DC voltage was recorded. The measurements were then utilized to calculate the overall rectenna efficiency (Fig. 33). To achieve the far-field condition at the required operating frequency, the separation distance between the transmitting and receiving antennas is set to 170 cm. A multimeter is employed to measure the output DC voltage across the load resistor. An Anritsu MG3697C RF signal generator was used to feed a wideband horn antenna. The output of the rectenna system was recorded using a digital multimeter, as illustrated in (Fig. 34), where the output DC is found to be 750 mV at RF received power of −3.7 dBm and transmitted power of 2 dBm at 4.2 GHz and R_L_=2500Ω. The results were recorded at various frequencies, and different levels of RF received power from the CP LWA antenna. Figure 35a illustrates the variation in efficiency versus RF received power using 2500 Ohm. The maximum efficiency was achieved at 0 dBm RF received power. Figure 35b shows the variation of measured DC output Voltage versus received power levels at different far field distances. The proposed rectenna exhibits high and stable efficiency across the frequency range of 4.1–4.43 GHz and 4.8–5.2 GHz, covering WLAN, WiMAX, and expected 5G mid-bands. Its maximum efficiency reaches 53.8% at 4.2 GHz with a received RF power of 0 dBm. Table 4 lists a comparison of the proposed leaky wave antenna with various proposed antennas. Table 5 presents a comparison of the proposed rectenna system with previously published systems. It can be noticed that the proposed LWA has a wide bandwidth, high gain correlated with circular polarization, which makes it suitable for RF EH application and achieving a high conversion efficiency value.

7 $$\:{P}_{outMax}=\:\frac{{{V}_{outMax}}^{2}}{{R}_{L}}=\:\frac{{{V}_{br}}^{2}}{{4R}_{L}}$$

8 $$\:PCE=\:\frac{{{V}_{out}}^{2}}{{R}_{L}\times\:\:{P}_{in}}$$

**Table 3:** The optimized dimensions of the wideband rectifier.

Components	Dimensions (mm)	Dimensions (mm)
TL_9_	L_9_ = 5	W_9_ = 5.5
TL_10_	L_10_ = 3	W_10_ = 0.7
TL_11_	L_11_ = 4	W_11_ = 1
TL_12_	L_12_ = 1.2	W_12_ = 2.8
TL_13_	L_13_ = 4	W_13_ = 1
TL_14_	L_14_ = 1.9	W_14_ = 1
TL_16_	L_16_ = 3	W_16_ = 1

**Table 4:** Comparison of the proposed CP LWA array with previously reported works.

Ref	Frequency (GHz)	Antenna size (mm3)	Antenna area relative to wavelength (λ)	Antenna polarization	Maximum gain (dBi)	Beam scanning/scanning angle	Antenna substrate
^54^	3–7	328 × 70 × 1	10.546λ × 2.250λ	-	14.1 and 13.9	32◦ to 13◦/ −22◦ to 21◦	F4B
^34^	5–11	320 × 55.56 × 0.5	13.793λ × 2.394λ	-	9.8	55⁰	Flexible dielectric film
^59^	9.8–15.5	18 × 139 × 1	0.23λ × 6.0λ × 0.04λ	Circular polarization	15.5	-52⁰ to 20⁰	F4BK
^60^	7–12	246.5 × 25.54 × 0.5	12.69λ × 1.31λ × 0.02λ	N/A	9.8	84⁰	F4B
^61^	6–16	N/A	5.7λ × 2.56λ	N/A	18.5	91⁰	F4B
^62^	5.8–9.4	N/A	6.68λ	N/A	14.6	-90⁰ to 90⁰	Rogers 4350
^44^	1–6	325 × 129.4 × 0.787	6.6422λ × 2.2557λ × 0.015	N/A	9.65	−35°and + 33°/−45°and + 29°	RogerRT5880
This work	2–8	297 × 70 × 1.52	8.918λ × 2.102λ	Circular polarization	9.8	-21⁰ to 29⁰	Rogers Ro4003C

**Table 5:** Comparison of the proposed rectenna with previously reported works.

Ref	Frequency (GHz)	Angular coverage	Input power level	Conversion efficiency	Area (mm^2^)	Diode	Vout (V)
^63^	1.8–2.6	Omnidirectional	7 dBm	50	140 × 140	SMS7630-079 L	1
^64^	1–5	Directional	9 dBm	> 30	58 × 55	SMS7630	N/A
^65^	2.45	Bidirectional	-10 dBm	N/A	N/A	SMS7630	1.7
^66^	2.45	Omnidirectional	0 dBm	65.1	98 × 100	SMS7630-005LF	1.65
^67^	3.5, 6, 8, 10, 18	Omnidirectional	0 dBm	25%	72 × 40	ZnO Schottky barrier	0.25
^68^	1.85, 2.25, 2.6, 3.52, 5.01, and 5.89	Omnidirectional	-10 dBm	43/41/41/36/22/19	N/A	SMS7630	0.26/0.25/0.25/0.23/0.18/0.17
^69^	3.5, 4.9, 5.8	Directional	0 dBm	51	N/A	SMS7630-079LF	0.99
^70^	2–18	Directional	0 dBm	40	N/A	HSMS-2850	3.6
This work	4–6	Directional with beam scanning	0 dBm/ -5dBm	53.8/ 43.2	376.5 × 103.3	SMS7630-079LF	1.2

## Conclusion

This paper proposed a circular polarized high gain and wide beam scanning rectenna. A spoof transmission line is designed for exciting the design of the antenna array. The STL has two rows of crescent patches on both sides is placed on a grounded dielectric substrate. The ground plane is grooved with circular slots. This design enhances the antenna’s impedance bandwidth, gain, and axial ratio performance. The antenna operates efficiently with a wide impedance bandwidth 120% (2–8 GHz) and axial ratio within frequency range 4.2–4.9 GHz and 5.4–5.5 GHz. The proposed design is fabricated, and characterized. The CP LWA array and rectifier are fully integrated to form the rectenna. The rectifier has been designed using a wideband resistance compression network (WRCN) technique to enhance the conversion efficiency. This approach has significantly improved conversion efficiency. Remarkably, power conversion efficiency (PCE) exceeding 30% has been achieved over a wide range of input power levels, spanning from-10 dBm to 10 dBm. The rectenna can provide a 50⁰ angular coverage, an average output voltage of 1.1 V, and an average RF-to-DC PCE of 47.5% at an input power of 0 dBm.

## Data Availability

The datasets used and/or analysed during the current study available from the corresponding author on reasonable request.
